# Tandem mass tag-based quantitative proteomic analysis of effects of multiple sevoflurane exposures on the cerebral cortex of neonatal and adult mice

**DOI:** 10.3389/fneur.2022.1056947

**Published:** 2022-12-13

**Authors:** Jingyu Feng, Hua Lin, Yue Zhao, Yongyan Yang, Xiaoli Zhuang, Yang Yu, Yonghao Yu

**Affiliations:** ^1^Department of Anesthesiology, Tianjin Medical University General Hospital, Tianjin, China; ^2^Tianjin Institute of Anesthesiology, Tianjin, China

**Keywords:** sevoflurane, developing brain, neurotoxicity, TMT-based quantitative proteomic analysis, anesthetic toxicity

## Abstract

**Introduction:**

Sevoflurane is the most commonly used general anesthetic in pediatric surgery, but it has the potential to be neurotoxic. Previous research found that long-term or multiple sevoflurane exposures could cause cognitive deficits in newborn mice but not adult mice, whereas short-term or single inhalations had little effect on cognitive function at both ages. The mechanisms behind these effects, however, are unclear.

**Methods:**

In the current study, 6- and 60-day-old C57bl mice in the sevoflurane groups were given 3% sevoflurane plus 60% oxygen for three consecutive days, each lasting 2 hours, while those in the control group only got 60% oxygen. The cortex tissues were harvested on the 8th or 62nd day. The tandem mass tags (TMT)pro-based quantitative proteomics combined with liquid chromatography-tandem mass spectrometry (LC-MS/MS) analysis, Golgi staining, and western blotting analysis were applied to analyze the influences of multiple sevoflurane anesthesia on the cerebral cortex in mice with various ages. The Morris water maze (MWM) test was performed from postnatal day (P)30 to P36 or P84 to P90 after control or multiple sevoflurane treatment. Sevoflurane anesthesia affected spatial learning and memory and diminished dendritic spines primarily in newborn mice, whereas mature animals exhibited no significant alterations.

**Results:**

A total of 6247 proteins were measured using the combined quantitative proteomics methods of TMTpro-labeled and LC-MS/MS, 443 of which were associated to the age-dependent neurotoxic mechanism of repeated sevoflurane anesthesia. Furthermore, western blotting research revealed that sevoflurane-induced brain damage in newborn mice may be mediated by increasing the levels of protein expression of CHGB, PTEN, MAP2c, or decreasing the level of SOD2 protein expression.

**Conclusion:**

Our findings would help to further the mechanistic study of age-dependent anesthetic neurotoxicity and contribute to seek for effective protection in the developing brain under general anesthesia.

## 1. Introduction

With rapid advances in anesthetic technology, millions of newborns and children worldwide undergo surgical interventions using general anesthesia, rendering child safety a major public health concern (1). Sevoflurane is the most commonly employed inhalational anesthetic in pediatric surgery (2). It has been reported that repeated or long-term sevoflurane exposure prior to 3–4 years of age can increase the potential for future learning and memory challenges (3–5), although available data remain debatable (6). Furthermore, our previous studies have demonstrated that multiple exposures to inhalational anesthetics, such as sevoflurane, can cause adverse effects, including neuroinflammation, apoptosis, synaptic insufficiency, and cognitive deficits in 6-day-old newborn mice, while 60-day-old adult mice showed no notable damage (7–11). The mechanisms underlying these age-dependent effects remain elusive.

Quantitative proteomics is a precise method for identifying differentially expressed proteins (DEPs) in biological processes or diseases and predicting therapeutic drug targets and underlying mechanisms (12). The continual development and application of molecular-based technologies have allowed researchers to explore the features of complex regulatory systems (13). Tandem mass tag (TMT)pro-based quantitative proteomics, a quantitative proteomics method, allows a large number of samples to be identified concurrently, thereby reducing batch effects (14). To further investigate the mechanism of age-dependent anesthetic neurotoxicity induced by sevoflurane, we used a combination of TMTpro-labeled quantitative proteomics and liquid chromatography-tandem mass spectrometry (LC-MS/MS) to identify DEPs after multiple inhalations of 3% sevoflurane plus 60% oxygen or 60% oxygen alone in neonatal and adult mice.

## 2. Materials and methods

### 2.1. Animals and experimental design

**Figure 1:**
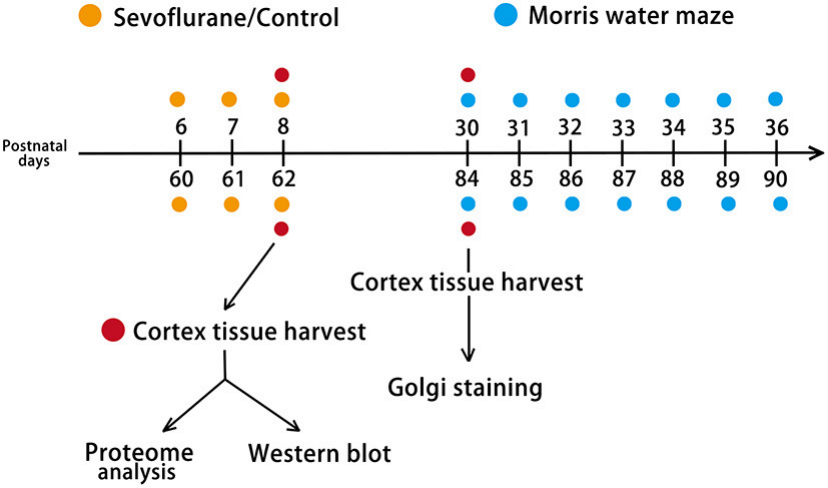
Experimental design. Mice were randomly assigned to four groups: P6 + Control, P6 + Sevoflurane, P60 + Control, and P60 + Sevoflurane. P6 (female plus male) and P60 (female) mice were subjected to 60% O_2_ + 3% sevoflurane or 60% O_2_ inhalation for 2 h over 3 consecutive days. Two hours after the last treatment, the cerebral cortex was harvested for TMT-based quantitative proteome analysis and western blotting. After 22 days of treatment, female mice were selected for the Morris water maze (MWM) test and Golgi staining. P6, postnatal day 6; P60, postnatal day 60; TMT, Tandem mass tag.

Pregnant mice (gestation days 16–17) and 60-day-old female C57BL/6J mice were purchased from Sibeifu Bioscience Company (license number, SCXK 2019-0010; Beijing, China). The mice were housed under 12 h of natural light and 12 h of darkness at a constant temperature (23 ± 1°C), with unrestricted access to food and water. Female and male newborn mouse pups delivered by pregnant mice were selected for experiments, including proteome analysis and western blotting, whereas only neonatal female mice were selected to undergo behavioral testing and Golgi staining. Mice were randomly allocated to postnatal day (P) 6 + Control, P6 + Sevoflurane, P60 + Control, and P60 + Sevoflurane groups. Twenty mice (*n* = 5 mice/group) were used for cerebral cortex proteomic analysis, 40 were subjected to the Morris water maze (MWM) test (*n* = 10 mice/group), 12 underwent Golgi staining (*n* = 3 mice/group), and 20 were used for western blotting analysis (*n* = 5 mice/group; Figure 1). All experiments were approved by the Animal Experimental Ethics Committee of Tianjin Medical University General Hospital in Tianjin, China (Approval No. IRB2021-DWFL-210). Every effort was made to reduce the suffering of mice and the number of animals used.

### 2.2. Multiple sevoflurane exposures and treatment

As described in our previous study (8), mice in the sevoflurane group were anesthetized using 3% sevoflurane and 60% oxygen at a flow rate of 2 L/min for 2 h per day for three consecutive days; the control group inhalationally administered 60% O_2_. Mice were exposed to sevoflurane in a transparent chamber (25 × 15 × 10 cm) connected with a sevoflurane-specific vaporizer. During treatment, the sevoflurane concentration was monitored using a gas monitor (Vamos/Vamos Plus; Draeger, Germany). The rectal temperature of mice was maintained at 37 ± 0.5°C using a heating blanket.

### 2.3. MWM

MWM trials were performed as described in our previous study (9). A circular pool (120 cm diameter × 60 cm height) was filled with opaque water using ~0.5 g/L titanium dioxide powder. The hidden white platform (diameter, 10 cm) was positioned in the center of the first quadrant, submerged 1.0 cm below the water surface. The water temperature in the pool was maintained at 23 ± 1°C. We tested P30 and P84 mice for 7 days (P30–P36; P84–P90), with four trials performed daily in the morning (from 8 to 12 am). The swimming behavior of mice was monitored using an automatic tracking system (Xinruan Information Technology Co. Ltd, Shanghai, China). On successfully locating the hidden platform within 90 s, the stopwatch was automatically stopped, and mice were maintained on the platform for 10 s. If a mouse failed to find the platform within 90 s, the animal was manually guided to the platform and then maintained on the platform for 15 s. To determine the spatial learning and memory performance of mice, we analyzed data related to escape latency during the training stage and platform crossing times in the probe test on P36 or P90. Mice were allowed a 30 min rest interval between each trial for daily training. After each training session, a heating pad was used to maintain a stable body temperature for 10 min.

### 2.4. Golgi staining and dendritic spine density analysis

The dendritic spine density of cortical neurons was identified using the FD Rapid GolgiStain Kit (Cat#: PK401, FD Neuro Technologies, USA). Mice were well-sedated for 4 min with 3% sevoflurane before decapitation on P30 or P84. Brains were subsequently dissected and immersed in a solution of mercuric chloride, potassium dichromate, and potassium chromate at room temperature for 14 days in the dark. The mixed solution was replaced the next day. Brain tissues were transferred to Solution C at room temperature in the dark for 96–120 h, and Solution C was replaced the following day. The impregnated brains were embedded in 4% agarose and sectioned at 120–150 μm using a Leica Vibratome. The slides were immersed in solutions D and E and Milli-Q water mixes. After careful washing with Milli-Q water, the slices were dehydrated with graded alcohol (50, 75, 95, and 100% ethanol in Milli-Q water) and cleared with xylene. Finally, the slides were sealed with neutral gum and analyzed under a bright field using a Nikon Eclipse TE2000U microscope. Ten pyramidal neurons from each mouse were selected in layers 4/5 of the cerebral cortex, and the dendritic spine density was counted using 10 secondary or third intermediate dendrites of 10-μm length under an oil immersion 100× objective. Thirty dendritic segments from each group were quantified using ImageJ2 (version 2.3, National Institutes of Health, USA). Dendritic spine density was calculated and expressed as the mean number of spines per 10 μm dendritic length.

### 2.5. Sample collection

Twenty mice were briefly anesthetized with sevoflurane 2 h after the end of treatment and decapitated. The cerebral cortex of each mouse was collected in independent cryogenic vials and stored in liquid nitrogen for rapid freezing. Samples were stored at −80°C until subsequent analysis. We selected TMTpro (16plek) based on 20 samples to reduce the loss of quantification data and counts of experimental batches. Two additional internal standards (IS) were introduced into our experiment as an independent group to reduce the impact of various batches. Both IS were mixtures of TMTpro-labeled peptides from 20 samples. Finally, TMT-based quantitative proteomic analysis with five biological replicates was performed on six groups of 22 samples.

### 2.6. Cortex homogenization and protein digestion

All tissue samples were homogenized on ice using lysis buffer (4% sodium dodecyl sulfate (SDS), 1 mM DTT, 100 mM Tris-HCl [pH 7.6], and protease inhibitor cocktail), and proteins were measured using the BCA protein assay kit (Bio-Rad Laboratories, USA). Briefly, samples were combined with 30 l SDT buffer (4% SDS, 100 mM DTT, 150 mM Tris-HCl, pH 8.0) for protein digestion. The detergent and DTT were subsequently removed using a mixture of uric acid (UA) buffer (8 M urea, 150 mM Tris-HCl, pH 8.0) and repeated ultrafiltration (Microcon units, 10 kD). Iodoacetamide [IAA; 100 μL iodoacetamide (100 mM IAA in UA buffer)] was added to the samples to block reduced cysteine residues, and samples were incubated in the dark for 30 min. The filters were then thrice washed with 100 μL UA buffer and twice with 100 μL 25 mM NH_4_HCO_3_ buffer. The peptides were collected as a filtrate after digestion with 4 g trypsin (Promega) in 40 μL 25 mM NH_4_HCO_3_ buffer overnight at 37°C. The peptides were desalted using C18 Cartridges [Sigma-Aldrich, USA, EmporeTM SPE Cartridges C18 (standard density) bed I.D. 7 mm, volume 3 mL], vacuum centrifuged, and reconstituted in 40 μL of 0.1% (v/v) formic acid. The extinction coefficient of 1.1 in the 0.1% (g/l) solution was calculated by considering the frequency of tryptophan and tyrosine in vertebrate proteins to estimate the ultraviolet (UV) light spectral density of peptides at 280 nm.

### 2.7. TMT labeling

For each sample, 100 μg of the peptide mixture was labeled using the TMTpro 16plek label reagent (Cat#: A44520, Thermo Fisher Scientific, USA) according to the manufacturer's instructions. The experiments were conducted in two batches. One batch comprised a set of peptides from the control group (10 samples) and IS, while the other consisted of peptides from sevoflurane groups (10 samples) and IS. The values of each batch were corrected using batch-specific IS. The samples were labeled using 11 of 16 TMTpro labels, including TMTpro-126, TMTpro-127C, TMTpro-127N, TMTpro-128C, TMTpro-128N, TMTpro-129C, TMTpro-129N, TMTpro-130C, TMTpro-130N, TMTpro-131C, and TMTpro-131N.

### 2.8. High-pH reversed-phase (RP) fractionation

Labeled peptides were fractionated to increase proteome coverage using a high-pH RP Peptide Fractionation Kit (Cat#:84868, Thermo Fisher Scientific, USA). The peptides were vacuum-dried prior to acidification with 0.1% trifluoroacetic acid (TFA) solution, followed by loading onto an equilibrated, high-pH, RP fractionation spin column. Peptides were bound to the hydrophobic resin under aqueous conditions and then desalted by washing the column with water using low-speed centrifugation at 5,000 × *g* for 2 min. After removing the solution and packing the resin, the liquid was discarded. To elute bound peptides into 10 separate fractions, a stepwise gradient of increasing acetonitrile (ACN) concentrations in a volatile high-pH elution solution was applied to columns recovered by centrifugation. The recovered fractions were desalted on C18 Cartridges (Cat#: EmporeTM SPE Cartridges C18 (standard density) bed I.D. 7 mm, volume 3 ml, Sigma-Aldrich, MO, USA) and vacuum centrifuged. The lyophilized peptide was re-dissolved in 12 μL of 0.1% formic acid solution (FA). The peptide concentration was measured at an optical density of 280 nm (OD280).

### 2.9. LC-MS/MS analysis

After loading into a reverse-phase trap column (Thermo Fisher Scientific, 100 m 2 cm) linked to a C18 RP analytical column (10 cm, ID75 μm, 3 μm resin), lyophilized peptides were resuspended in buffer A (0.1% FA). The tagged peptides were separated using an IntelliFlow-controlled linear gradient of buffer B (84% ACN and 0.1% FA) at a flow rate of 30 NL/min.

For LC-MS/MS analysis, we used a Q-Exactive mass spectrometer (Thermo Fisher Scientific) and an Easy nLC system. MS data were collected at 70,000 resolutions and 200 m/z in a scan range of 300–1,800 m/z in positive ion mode to detect intact peptides. The AGC goal was set at 1e6, the maximum inject time was 10 ms, and the dynamic exclusion duration was 40.0 s. In total, 20 MS2 scans were obtained based on the following settings to capture the mass-charge ratios of the polypeptide and polypeptide fragments for each entire scan: MS2 was activated with HCD, the isolation window was set to 2 m/z, the resolution of the HCD spectra was set to 17,500 at 200 m/z, the normalized collision energy was 30 eV, and the underfill ratio was 0.1%.

### 2.10. Protein identification and quantification

The raw data for each sample were processed using Proteome Discover software (version 1.4, Thermo Fisher Scientific) and the MASCOT engine (version 2.2, Matrix Science, London, UK) to match with the Uniport mouse database (“Swissprot_Mus_musculus_17063_20210106.fasta” downloaded from^1^ on January 6, 2021, and included 17,063 protein sequences). The following search parameters were entered: Mas missed cleavages: 2; peptide mass tolerance: ± 20 ppm; fragment mass tolerance: 0.1 Da; enzyme: Trypsin; Carbamidomethyl (C), TMT 6/10/16 plex (N-term), TMT 6/10/16 plex (K) are fixed modifications, and oxidation (M) is a variable modification. Decoy is a database pattern. Effective peptides had a false discovery rate of <0.01. Protein ratios were determined as the median of distinct peptides used to measure proteins. To account for experimental bias, all protein ratios were standardized using the mean protein ratio. The mass spectrometry proteomics data have been deposited to the ProteomeXchange Consortium *via* the PRIDE partner repository with the dataset identifier PXD037294.

### 2.11. Western blot

On completing the sevoflurane treatment, mice were decapitated, and the cerebral cortex was extracted after short-term anesthesia with 3% sevoflurane for 5 min. RIPA buffer (Cat#: R0020, Solarbio, China) and protease inhibitors (Cat#: HY-K0010, MedChemExpress, China) were used for tissue lysis. The extracted proteins were examined to assess the expression of the following proteins and confirm proteomic data: chromogranin B (CHGB), secretogranin-2 (SCG2), phosphatase and tensin homolog (PTEN), microtubule-associated protein 2c (MAP2c), and mitochondrial superoxide dismutase 2 (MSOD2) (SOD2). The protein concentration was measured using a BCA protein assay kit (Cat#: CW0014S, CWBIO, China). Identical protein amounts were loaded onto 4–12% SDS- polyacrylamide gel electrophoresis gels (Cat#: M00652, Gennscript, China), and proteins were transferred to polyvinylidene fluoride membranes (Cat#: IPVH00010, Millipore, US) and blocked with SuperBlock (TBS) Blocking Buffer (Cat#:37536, Thermo Fisher Scientific, USA) for 30 min. Membranes were incubated with the following primary antibodies: CHGB (1:1000; Cat#: ab150354, Abcam, UK), SCG2 (1:800; Cat#: NO.20357-1-AP, ProteinTech, USA), PTEN (1:100; Santa Cruz Biotechnology, USA), MAP2 (1:1000; Cat#: NO. 17490-1-AP, Proteintech, USA), SCG2 (1:1000; Cat#: NO. 24127-1-AP, ProteinTech, USA), and GAPDH (1:5000; Cat#: T0004, Affinity Biosciences, USA) at 4°C overnight. After washing five times with TBST (1× Tris-buffered saline with 0.1% Tween-20), membranes were then incubated with the horseradish peroxidase (HRP)-conjugated goat anti-mouse (1:5000; Cat#: S0002 Affinity Biosciences, USA) or rabbit secondary antibody (1:5000; Cat#: S0001 Affinity Biosciences, USA) at room temperature for 1.5 h, following washing mentioned above. Finally, the protein bands were visualized with ECL solution, and after obtaining images, the primary and secondary antibodies of the membrane were stripped by western blot stripping buffer (Cat#: 21059, ThermoFisher Scientific, USA) for 30–60 min at 37°C, depending on antibody affinity. The next target protein was incubated on the stripped membrane with primary and secondary antibodies, as described previously. ImageJ2 was used to qualify the protein bands (version 2.3, National Institutes of Health, USA). The protein expression levels of CHGB, SCG2, MAP2c, PTEN, and SOD2 were normalized to GAPDH levels. The experiment was performed in triplicate.

### 2.12. Statistical analysis

Data analyses were performed using GraphPad Prism version 9.0 (GraphPad Software Inc., La Jolla, CA, USA). The results of biochemical experiments are presented as mean ± standard deviation (SD). Values of MWM escape latency are presented as the mean ± standard error of the mean (SEM), and platform crossing numbers are expressed as the median and interquartile range.

A normality test was used to determine whether data values were normally distributed. Two-way repeated-measures ANOVA was used to compare escape latency in behavioral data. The Mann-Whitney test was used to assess the number of platform crossings between the control and sevoflurane anesthesia groups. Two-way ANOVA was performed to examine the influence of age (6-day-old vs. 60-day-old), treatment (control *vs*. sevoflurane treatment), and the interaction between age and treatment on protein expression levels (CHGB, PTEN, MAP2c, and SOD2). One-way ANOVA was used to examine differences in the relative density of the proteins described above, as well as the density of dendritic spines. *P* < 0.05 was deemed as a statistically significant difference.

## 3. Results

### 3.1. Multiple sevoflurane inhalations induce future spatial learning and memory deficits in newborn but not in adult mice

**Figure 2:**
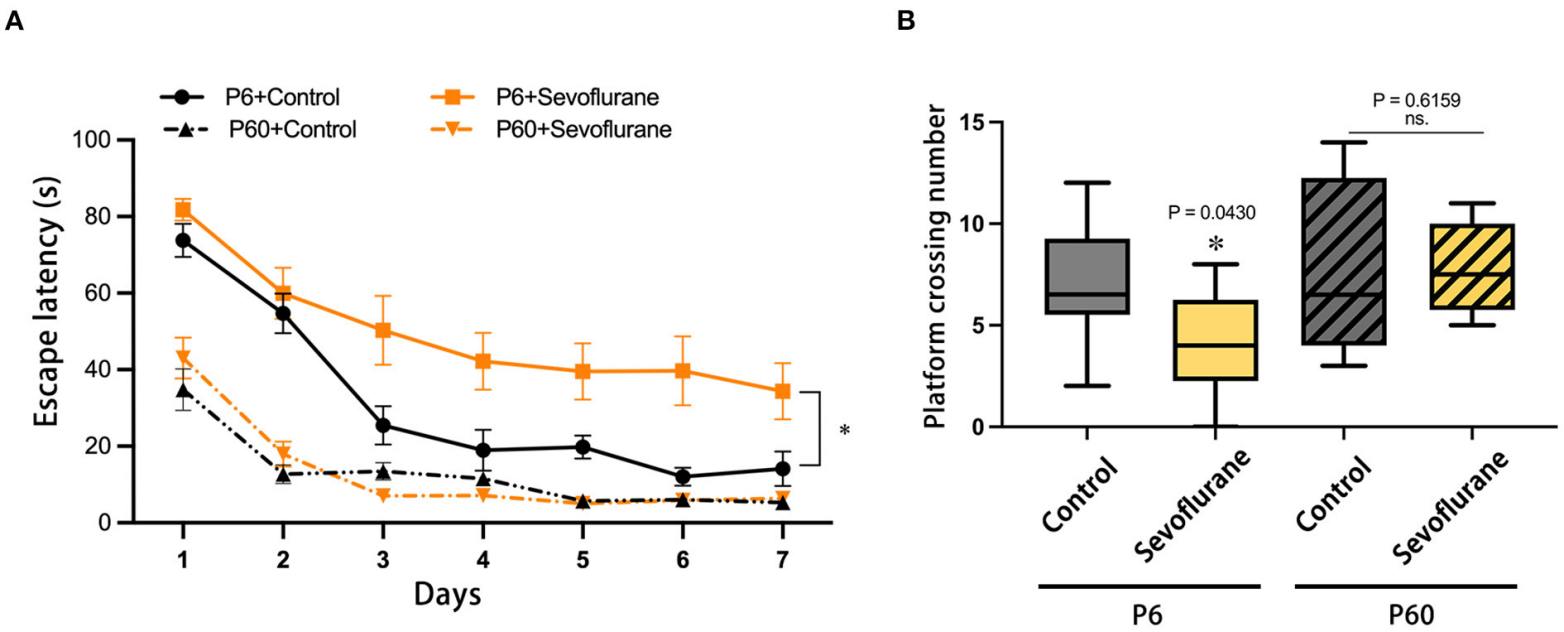
Effects of multiple exposures to sevoflurane anesthesia to neonatal and adult mice on future spatial cognitive function in the test. **(A)** Swimming escape latency and **(B)** platform crossing number. *n* = 10 mice/group. Escape latency results are presented as mean ± standard error of the mean (SEM); the platform crossing number is expressed as median and interquartile range. *P < 0.05 vs. P6 + Control. MWM, Morris water maze; P6, postnatal day 6.

Data on escape latency and platform crossing numbers were collected throughout the positional navigation training and spatial exploration assessments. Considering the escape latency in the positioning navigation stage, pubertal mice subjected to multiple 3% sevoflurane anesthesia throughout neonatal development exhibited impaired spatial learning (*P* < 0.05), with no statistical difference observed between the P60 + Control and P60 + Sevoflurane groups (Figure 2A). Furthermore, sevoflurane anesthesia decreased the platform crossing number of adolescent mice when compared with that of control mice (*P* < 0.05), with no notable difference observed in the adult groups (Figure 2B). These results suggested that multiple sevoflurane doses could impair the spatial learning and memory function of newborn mice during puberty, with no significant effect observed in adult mice.

### 3.2. Changes in dendritic spine density in cerebral cortex

**Figure 3:**
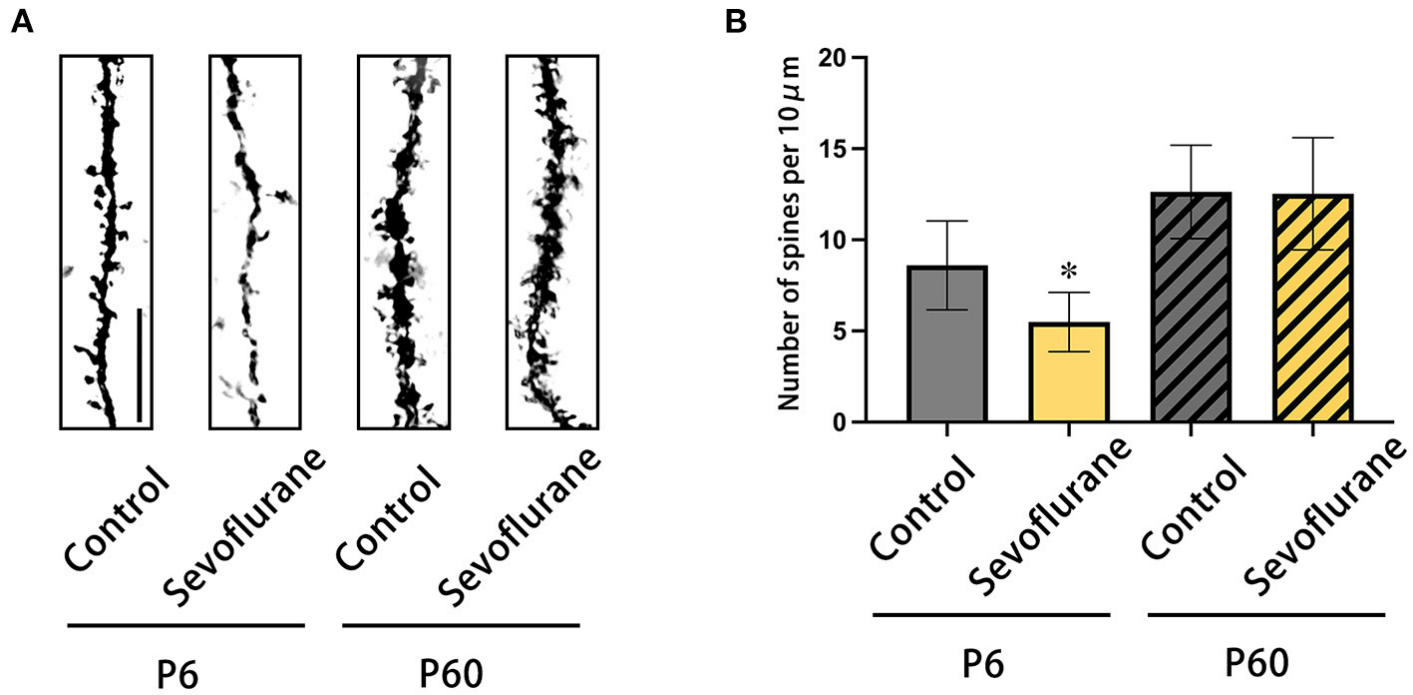
The results of Golgi-Cox staining on the dendrites of pyramidal neurons of cerebral cortex in layers 4/5. **(A)** Representative photomicrographs of Golgi-Cox-stained dendrites (scale bar, 1 μm). **(B)** The dendritic spine density (numbers of dendritic spine/10 μm). *n* = 20 dendrites/mice. *P < 0.05 vs. P6 + Control. P6, postnatal day 6.

Based on Golgi-Cox staining of the cerebral cortex harvested 22 days post-treatment, pyramidal neurons in layer 4/5 of the P6+Sevoflurane group had a lower dendritic spine density than those of the P6+Control group; multiple sevoflurane exposures did not affect future dendritic spine density in adult mice. The above results were compatible with the behavioral test results (^*^P <0.05, P6 + Control, Figure 3).

### 3.3. Protein quantitative characterization and DEP screening

A TMT-based quantitative proteomics technique was used to examine the cerebral cortex proteome of different groups. Our findings indicated that 6,861 proteins were discovered, with 6,247 proteins presenting quantitative values and annotation words. In the present study, proteins whose quantitative levels increased or decreased 1.2-fold were deemed DEPs.

Four groups were used for pairwise comparisons. DEPs derived from sevoflurane-induced neurotoxicity in newborn mice met at least one of three criteria: (1) in neonatal mice, sevoflurane caused the differential expression of proteins (DEPs in the P6 + Sevoflurane/P6 + Control), while age induced differential protein expression in control group mice (DEPs in the P60 + Control/P6 + Control). Discrepancies in DEP changes between the two groups implied that sevoflurane anesthesia might suppress age-induced DEPs, therefore promoting neurotoxic vulnerability in young mice (Table 1); (2) sevoflurane caused DEPs in newborn groups (DEPs in the P6 + Sevoflurane/P6 + Control), but age had no effect on the expression of these proteins in the sevoflurane treatment group (non-DEPs in the P60 + Sevoflurane/P6 + Sevoflurane). This finding suggests that these proteins could be intervention targets for sevoflurane-induced developmental neurotoxicity and are not associated with age. The therapeutic potential of these proteins could be exploited using selective inhibitors to prevent damage associated with multiple sevoflurane inhalations at a young age (Table 2); (3) Multiple sevoflurane inhalation induced statistically significant differences in protein expression in the neonatal group (DEPs in the P6 + Sevoflurane/P6 + Control), whereas age could also induce these proteins to produce significant differential expression in the sevoflurane group (DEPs in the P60 + Sevoflurane/P6 + Sevoflurane group). This finding revealed that targets of sevoflurane-induced neurotoxicity in neonatal mice matched those of age-dependent DEPs exposed to several sevoflurane doses (Table 3). Based on the above criteria, 443 proteins were filtered out to evaluate the potential mechanisms of sevoflurane-induced developmental neurotoxicity.

**Table 1:** List of the several sevoflurane-related significantly differential proteins in condition 1.

**Protein names**	**Proteins IDs**	**Gene name**	**P6S/P6C**	***P***	**P60S/6S**	***P***
Phospholemman	Q9Z239	Fxyd1	2.83	*P* < 0.05	0.68	*P* < 0.05
Intersectin-2	Q9Z0R6	Itsn2	1.41	*P* < 0.05	0.69	*P* < 0.05
NPC intracellular cholesterol transporter 2	Q9Z0J0	Npc2	0.78	*P* < 0.05	1.22	*P* < 0.05
A-kinase anchor protein 12	Q9WTQ5	Akap12	0.74	*P* < 0.05	1.34	*P* < 0.05
KH domain-containing, RNA-binding, signal transduction-associated protein 3	Q9R226	Khdrbs3	0.76	*P* < 0.05	1.51	*P* < 0.05
Serine racemase	Q9QZX7	Srr	1.30	*P* < 0.05	0.66	*P* < 0.05
Tubulin alpha-8 chain	Q9JJZ2	Tuba8	1.47	*P* < 0.05	0.58	*P* < 0.05
Transcription and mRNA export factor ENY2	Q9JIX0	Eny2	0.81	*P* < 0.05	1.32	*P* < 0.05
Fructosamine-3-kinase	Q9ER35	Fn3k	2.00	*P* < 0.05	0.83	*P* < 0.05
Transcription factor 20	Q9EPQ8	Tcf20	0.65	*P* < 0.05	1.64	*P* < 0.05
NADH dehydrogenase [ubiquinone] iron-sulfur protein 3, mitochondrial	Q9DCT2	Ndufs3	1.59	*P* < 0.05	0.83	*P* < 0.05
Methyltransferase-like 26	Q9DCS2	Mettl26	1.22	*P* < 0.05	0.69	*P* < 0.05
NADH dehydrogenase [ubiquinone] 1 alpha subcomplex subunit 9, mitochondrial	Q9DC69	Ndufa9	1.67	*P* < 0.05	0.83	*P* < 0.05
Ubiquitin carboxyl-terminal hydrolase 12	Q9D9M2	Usp12	1.53	*P* < 0.05	0.82	*P* < 0.05
Synaptojanin-2-binding protein	Q9D6K5	Synj2bp	1.54	*P* < 0.05	0.73	*P* < 0.05
1-acyl-sn-glycerol-3-phosphate acyltransferase gamma	Q9D517	Agpat3	1.25	*P* < 0.05	0.64	*P* < 0.05
Protein tweety homolog 1	Q9D3A9	Ttyh1	1.88	*P* < 0.05	0.82	*P* < 0.05
Heterogeneous nuclear ribonucleoprotein A0	Q9CX86	Hnrnpa0	0.57	*P* < 0.05	1.41	*P* < 0.05
ATP synthase subunit s, mitochondrial	Q9CRA7	Dmac2l	1.49	*P* < 0.05	0.78	*P* < 0.05
Josephin-2	Q9CR30	Josd2	2.44	*P* < 0.05	0.75	*P* < 0.05
Acetyl-coenzyme A transporter 1	Q99J27	Slc33a1	1.53	*P* < 0.05	0.68	*P* < 0.05
Calcium/calmodulin-dependent protein kinase type II subunit gamma	Q923T9	Camk2g	1.45	*P* < 0.05	0.83	*P* < 0.05
ADP-ribose glycohydrolase MACROD1	Q922B1	Macrod1	1.28	*P* < 0.05	0.80	*P* < 0.05
Prostaglandin reductase 2	Q8VDQ1	Ptgr2	1.50	*P* < 0.05	0.63	*P* < 0.05
Small glutamine-rich tetratricopeptide repeat-containing protein beta	Q8VD33	Sgtb	1.24	*P* < 0.05	0.73	*P* < 0.05
Cytochrome b-c1 complex subunit 9	Q8R1I1	Uqcr10	1.60	*P* < 0.05	0.59	*P* < 0.05
Synaptogyrin-3	Q8R191	Syngr3	1.31	*P* < 0.05	0.68	*P* < 0.05
ATP-binding cassette sub-family F member 3	Q8K268	Abcf3	0.82	*P* < 0.05	1.53	*P* < 0.05
COX assembly mitochondrial protein 2 homolog	Q8K199	Cmc2	1.20	*P* < 0.05	0.81	*P* < 0.05
Zinc finger protein 536	Q8K083	Znf536	0.52	*P* < 0.05	1.60	*P* < 0.05
Adhesion G protein-coupled receptor A1	Q8C4G9	Adgra1	1.42	*P* < 0.05	0.60	*P* < 0.05
Copine-1	Q8C166	Cpne1	1.29	*P* < 0.05	0.81	*P* < 0.05
Coronin-2A	Q8C0P5	Coro2a	1.45	*P* < 0.05	0.57	*P* < 0.05
Choline transporter-like protein 2	Q8BY89	Slc44a2	1.24	*P* < 0.05	0.79	*P* < 0.05
Uncharacterized protein KIAA1671	Q8BRV5	Kiaa1671	1.45	*P* < 0.05	0.81	*P* < 0.05
Ubiquinone biosynthesis O-methyltransferase, mitochondrial	Q8BMS4	Coq3	1.43	*P* < 0.05	0.75	*P* < 0.05
PI-PLC X domain-containing protein 3	Q8BLJ3	Plcxd3	1.26	*P* < 0.05	0.73	*P* < 0.05
Serine/arginine-rich splicing factor 7	Q8BL97	Srsf7	0.63	*P* < 0.05	1.21	*P* < 0.05
Potassium voltage-gated channel subfamily F member 1	Q7TSH7	Kcnf1	1.46	*P* < 0.05	0.71	*P* < 0.05
MICOS complex subunit Mic10	Q7TNS2	Micos10	1.60	*P* < 0.05	0.77	*P* < 0.05
WD repeat-containing protein 43	Q6ZQL4	Wdr43	0.81	*P* < 0.05	1.28	*P* < 0.05
FK506-binding protein 15	Q6P9Q6	Fkbp15	0.83	*P* < 0.05	1.21	*P* < 0.05
Structure-specific endonuclease subunit SLX4	Q6P1D7	Slx4	0.76	*P* < 0.05	1.30	*P* < 0.05
Centrosomal protein of 170 kDa	Q6A065	Cep170	0.74	*P* < 0.05	1.21	*P* < 0.05
Long-chain fatty acid transport protein 1	Q60714	Slc27a1	1.23	*P* < 0.05	0.80	*P* < 0.05
Laminin subunit alpha-2	Q60675	Lama2	1.29	*P* < 0.05	0.67	*P* < 0.05
Corticotropin-releasing factor-binding protein	Q60571	Crhbp	1.21	*P* < 0.05	0.77	*P* < 0.05
Leucine-rich repeat serine/threonine-protein kinase 2	Q5S006	Lrrk2	1.52	*P* < 0.05	0.67	*P* < 0.05
Extracellular serine/threonine protein kinase FAM20C	Q5MJS3	Fam20c	0.74	*P* < 0.05	1.25	*P* < 0.05
Zinc transporter ZIP12	Q5FWH7	Slc39a12	1.26	*P* < 0.05	0.65	*P* < 0.05
Sodium channel subunit beta-2	Q56A07	Scn2b	2.71	*P* < 0.05	0.49	*P* < 0.05
Optic atrophy 3 protein homolog	Q505D7	Opa3	1.25	*P* < 0.05	0.71	*P* < 0.05
BMP/retinoic acid-inducible neural-specific protein 3	Q499E0	Brinp3	1.21	*P* < 0.05	0.68	*P* < 0.05
Type 1 phosphatidylinositol 4,5-bisphosphate 4-phosphatase	Q3TWL2	Pip4p1	1.33	*P* < 0.05	0.74	*P* < 0.05
Coiled-coil domain-containing protein 127	Q3TC33	Ccdc127	1.49	*P* < 0.05	0.72	*P* < 0.05
Cyclin-dependent kinase 16	Q04735	Cdk16	1.41	*P* < 0.05	0.71	*P* < 0.05
Isochorismatase domain-containing protein 2A	P85094	Isoc2a	2.13	*P* < 0.05	0.74	*P* < 0.05
Cellular retinoic acid-binding protein 1	P62965	Crabp1	0.67	*P* < 0.05	1.34	*P* < 0.05
Mitochondrial import inner membrane translocase subunit Tim13	P62075	Timm13	1.25	*P* < 0.05	0.72	*P* < 0.05
Wolframin	P56695	Wfs1	1.44	*P* < 0.05	0.68	*P* < 0.05
Arylsulfatase A	P50428	Arsa	1.23	*P* < 0.05	0.77	*P* < 0.05
Tropomodulin-1	P49813	Tmod1	2.07	*P* < 0.05	0.64	*P* < 0.05
Glutamate decarboxylase 1	P48318	Gad1	1.43	*P* < 0.05	0.73	*P* < 0.05
Ras-specific guanine nucleotide-releasing factor 1	P27671	Rasgrf1	1.33	*P* < 0.05	0.67	*P* < 0.05
Splicing factor U2AF 65 kDa subunit	P26369	U2af2	0.61	*P* < 0.05	1.79	*P* < 0.05
Gap junction alpha-1 protein	P23242	Gja1	1.62	*P* < 0.05	0.74	*P* < 0.05
Cytochrome c oxidase subunit 7C, mitochondrial	P17665	Cox7c	1.42	*P* < 0.05	0.74	*P* < 0.05
Integrin beta-2	P11835	Itgb2	1.61	*P* < 0.05	0.52	*P* < 0.05
Superoxide dismutase [Mn], mitochondrial	P09671	Sod2	1.59	*P* < 0.05	0.74	*P* < 0.05
Major prion protein	P04925	Prnp	1.42	*P* < 0.05	0.76	*P* < 0.05
NADH-ubiquinone oxidoreductase chain 4	P03911	Mtnd4	1.28	*P* < 0.05	0.78	*P* < 0.05
Protein Wiz	O88286	Wiz	0.72	*P* < 0.05	1.27	*P* < 0.05
Transcription factor Sp3	O70494	Sp3	0.71	*P* < 0.05	1.23	*P* < 0.05
ATPase GET3	O54984	Get3	1.29	*P* < 0.05	0.70	*P* < 0.05
DnaJ homolog subfamily B member 6	O54946	Dnajb6	1.21	*P* < 0.05	0.73	*P* < 0.05
YjeF N-terminal domain-containing protein 3	F6W8I0	Yjefn3	1.78	*P* < 0.05	0.70	*P* < 0.05
Glutamate-rich protein 6	D3Z6S9	Erich6	1.34	*P* < 0.05	0.76	*P* < 0.05
Damage-control phosphatase ARMT1	A6H630	Armt1	1.48	*P* < 0.05	0.79	*P* < 0.05
Apical junction component 1 homolog	A2AJA9	Ajm1	1.54	*P* < 0.05	0.75	*P* < 0.05
Oxysterol-binding protein-related protein 9	A2A8Z1	Osbpl9	0.79	*P* < 0.05	1.29	*P* < 0.05

**Table 2:** List of the several sevoflurane-related significantly differential proteins in condition 2.

**Protein names**	**Proteins IDs**	**Gene name**	**P6S/P6C**	***P***	**P60S/6S**	***P***
Oxysterol-binding protein-related protein 10	S4R1M9	Osbpl10	0.79	*P* < 0.05	0.85	*P* > 0.05
Protein sel-1 homolog 1	Q9Z2G6	Sel1l	0.82	*P* < 0.05	1.02	*P* > 0.05
Protein fem-1 homolog A-A	Q9Z2G1	Fem1aa	0.80	*P* < 0.05	1.07	*P* > 0.05
Disintegrin and metalloproteinase domain-containing protein 17	Q9Z0F8	Adam17	1.28	*P* < 0.05	1.06	*P* > 0.05
Beta-crystallin B1	Q9WVJ5	Crybb1	0.82	*P* < 0.05	0.93	*P* > 0.05
Ribosomal protein S6 kinase alpha-2	Q9WUT3	Rps6ka2	0.78	*P* < 0.05	1.01	*P* > 0.05
YLP motif-containing protein 1	Q9R0I7	Ylpm1	1.21	*P* < 0.05	0.97	*P* > 0.05
Evolutionarily conserved signaling intermediate in Toll pathway, mitochondrial	Q9QZH6	Ecsit	0.81	*P* < 0.05	0.99	*P* > 0.05
DCN1-like protein 1	Q9QZ73	Dcun1d1	0.74	*P* < 0.05	0.92	*P* > 0.05
ProSAAS	Q9QXV0	Pcsk1n	1.20	*P* < 0.05	1.06	*P* > 0.05
Amyloid-beta A4 precursor protein-binding family B member 1	Q9QXJ1	Apbb1	1.41	*P* < 0.05	0.90	*P* > 0.05
Trafficking protein particle complex subunit 2-like protein	Q9JME7	Trappc2l	0.82	*P* < 0.05	0.98	*P* > 0.05
VPS10 domain-containing receptor SorCS1	Q9JLC4	Sorcs1	1.26	*P* < 0.05	1.01	*P* > 0.05
CCR4-NOT transcription complex subunit 9	Q9JKY0	Cnot9	0.81	*P* < 0.05	0.92	*P* > 0.05
Syntaxin-6	Q9JKK1	Stx6	0.77	*P* < 0.05	0.89	*P* > 0.05
Kv channel-interacting protein 2	Q9JJ69	Kcnip2	0.71	*P* < 0.05	0.89	*P* > 0.05
Coatomer subunit beta	Q9JIF7	Copb1	0.77	*P* < 0.05	0.87	*P* > 0.05
Progressive ankylosis protein	Q9JHZ2	Ankh	1.28	*P* < 0.05	1.05	*P* > 0.05
Transcription factor 20	Q9EPQ8	Tcf20	1.64	*P* < 0.05	1.03	*P* > 0.05
X-linked retinitis pigmentosa GTPase regulator-interacting protein 1	Q9EPQ2	Rpgrip1	0.46	*P* < 0.05	0.83	*P* > 0.05
STARD3 N-terminal-like protein	Q9DCI3	Stard3nl	1.70	*P* < 0.05	0.88	*P* > 0.05
Protein-associating with the carboxyl-terminal domain of ezrin	Q9DBQ7	Scyl3	1.51	*P* < 0.05	0.98	*P* > 0.05
Vesicular integral-membrane protein VIP36	Q9DBH5	Lman2	0.80	*P* < 0.05	0.81	*P* > 0.05
Ubiquitin carboxyl-terminal hydrolase 12	Q9D9M2	Usp12	0.82	*P* < 0.05	1.11	*P* > 0.05
TP53-regulated inhibitor of apoptosis 1	Q9D8Z2	Triap1	0.74	*P* < 0.05	0.85	*P* > 0.05
SRA stem-loop-interacting RNA-binding protein, mitochondrial	Q9D8T7	Slirp	0.75	*P* < 0.05	0.92	*P* > 0.05
Protein FAM241B	Q9D882	Fam241b	1.32	*P* < 0.05	0.96	*P* > 0.05
Protein FAM162A	Q9D6U8	Fam162a	0.73	*P* < 0.05	0.91	*P* > 0.05
Clavesin-1	Q9D4C9	Clvs1	0.80	*P* < 0.05	1.02	*P* > 0.05
Epoxide hydrolase 1	Q9D379	Ephx1	0.71	*P* < 0.05	0.96	*P* > 0.05
Arpin	Q9D0A3	Arpin	1.37	*P* < 0.05	0.93	*P* > 0.05
Ras-related protein Rab-3B	Q9CZT8	Rab3b	0.82	*P* < 0.05	0.86	*P* > 0.05
Succinate dehydrogenase cytochrome b560 subunit, mitochondrial	Q9CZB0	Sdhc	0.78	*P* < 0.05	0.92	*P* > 0.05
Probable ATP-dependent RNA helicase DDX47	Q9CWX9	Ddx47	1.32	*P* < 0.05	0.99	*P* > 0.05
ATP synthase subunit s, mitochondrial	Q9CRA7	Dmac2l	0.78	*P* < 0.05	0.96	*P* > 0.05
Calcium-regulated heat stable protein 1	Q9CR86	Carhsp1	1.48	*P* < 0.05	1.01	*P* > 0.05
Transmembrane protein 33	Q9CR67	Tmem33	0.63	*P* < 0.05	0.97	*P* > 0.05
L-aminoadipate-semialdehyde dehydrogenase-phosphopantetheinyl transferase	Q9CQF6	Aasdhppt	0.55	*P* < 0.05	0.82	*P* > 0.05
Ubiquilin-4	Q99NB8	Ubqln4	1.57	*P* < 0.05	0.94	*P* > 0.05
39S ribosomal protein L9, mitochondrial	Q99N94	Mrpl9	1.65	*P* < 0.05	1.08	*P* > 0.05
39S ribosomal protein L16, mitochondrial	Q99N93	Mrpl16	0.77	*P* < 0.05	0.87	*P* > 0.05
RING finger protein 141	Q99MB7	Rnf141	0.81	*P* < 0.05	0.93	*P* > 0.05
CDK5 regulatory subunit-associated protein 3	Q99LM2	Cdk5rap3	2.06	*P* < 0.05	1.15	*P* > 0.05
Chloride channel CLIC-like protein 1	Q99LI2	Clcc1	1.35	*P* < 0.05	0.94	*P* > 0.05
Ras-related GTP-binding protein C	Q99K70	Rragc	0.80	*P* < 0.05	0.86	*P* > 0.05
Alsin	Q920R0	Als2	0.83	*P* < 0.05	0.93	*P* > 0.05
GTP-binding protein Di-Ras1	Q91Z61	Diras1	0.80	*P* < 0.05	1.00	*P* > 0.05
Thymocyte nuclear protein 1	Q91YJ3	Thyn1	0.76	*P* < 0.05	0.83	*P* > 0.05
Oxysterol-binding protein-related protein 1	Q91XL9	Osbpl1a	0.78	*P* < 0.05	0.88	*P* > 0.05
SNF-related serine/threonine-protein kinase	Q8VDU5	Snrk	1.21	*P* < 0.05	1.03	*P* > 0.05
Small glutamine-rich tetratricopeptide repeat-containing protein beta	Q8VD33	Sgtb	0.73	*P* < 0.05	0.88	*P* > 0.05
Alpha/beta hydrolase domain-containing protein 17C	Q8VCV1	Abhd17c	0.82	*P* < 0.05	0.93	*P* > 0.05
UBX domain-containing protein 4	Q8VCH8	Ubxn4	1.55	*P* < 0.05	0.93	*P* > 0.05
Netrin-G1	Q8R4G0	Ntng1	0.72	*P* < 0.05	0.84	*P* > 0.05
COMM domain-containing protein 5	Q8R395	Commd5	0.66	*P* < 0.05	1.12	*P* > 0.05
Rab11 family-interacting protein 5	Q8R361	Rab11fip5	1.24	*P* < 0.05	1.01	*P* > 0.05
Synaptogyrin-3	Q8R191	Syngr3	0.68	*P* < 0.05	0.87	*P* > 0.05
Optineurin	Q8K3K8	Optn	0.72	*P* < 0.05	0.95	*P* > 0.05
Netrin receptor UNC5A	Q8K1S4	Unc5a	1.78	*P* < 0.05	1.02	*P* > 0.05
COMM domain-containing protein 10	Q8JZY2	Commd10	1.27	*P* < 0.05	1.07	*P* > 0.05
Long-chain-fatty-acid–CoA ligase 5	Q8JZR0	Acsl5	0.78	*P* < 0.05	0.86	*P* > 0.05
TGF-beta-activated kinase 1 and MAP3K7-binding protein 1	Q8CF89	Tab1	0.71	*P* < 0.05	1.05	*P* > 0.05
von Willebrand factor A domain-containing protein 8	Q8CC88	Vwa8	0.80	*P* < 0.05	1.08	*P* > 0.05
Consortin	Q8CBC4	Cnst	0.71	*P* < 0.05	0.86	*P* > 0.05
Prenylcysteine oxidase-like	Q8C7K6	Pcyox1l	1.23	*P* < 0.05	1.00	*P* > 0.05
RNA-binding protein 14	Q8C2Q3	Rbm14	1.20	*P* < 0.05	0.95	*P* > 0.05
Copine-1	Q8C166	Cpne1	0.81	*P* < 0.05	0.98	*P* > 0.05
Autophagy-related protein 16-1	Q8C0J2	Atg16l1	0.83	*P* < 0.05	0.97	*P* > 0.05
Pogo transposable element with ZNF domain	Q8BZH4	Pogz	1.21	*P* < 0.05	0.95	*P* > 0.05
Calcium/calmodulin-dependent protein kinase type 1D	Q8BW96	Camk1d	0.80	*P* < 0.05	0.90	*P* > 0.05
Uncharacterized protein KIAA1671	Q8BRV5	Kiaa1671	0.81	*P* < 0.05	1.19	*P* > 0.05
Paralemmin-2	Q8BR92	Palm2	1.24	*P* < 0.05	1.06	*P* > 0.05
IQ calmodulin-binding motif-containing protein 1	Q8BP00	Iqcb1	0.77	*P* < 0.05	1.04	*P* > 0.05
PI-PLC X domain-containing protein 3	Q8BLJ3	Plcxd3	0.73	*P* < 0.05	0.89	*P* > 0.05
RNA binding protein fox-1 homolog 3	Q8BIF2	Rbfox3	0.75	*P* < 0.05	0.81	*P* > 0.05
Atlastin-1	Q8BH66	Atl1	0.83	*P* < 0.05	0.97	*P* > 0.05
Enolase-phosphatase E1	Q8BGB7	Enoph1	0.83	*P* < 0.05	0.90	*P* > 0.05
Protein ZNF365	Q8BG89	Znf365	0.71	*P* < 0.05	0.97	*P* > 0.05
Coiled-coil domain-containing protein 50	Q810U5	Ccdc50	0.72	*P* < 0.05	0.81	*P* > 0.05
Tetratricopeptide repeat protein 9C	Q810A3	Ttc9c	0.75	*P* < 0.05	0.99	*P* > 0.05
Sperm-associated antigen 1	Q80ZX8	Spag1	1.75	*P* < 0.05	0.99	*P* > 0.05
Death-associated protein kinase 1	Q80YE7	Dapk1	0.75	*P* < 0.05	1.04	*P* > 0.05
Rabenosyn-5	Q80Y56	Rbsn	0.77	*P* < 0.05	0.82	*P* > 0.05
MICOS complex subunit Mic10	Q7TNS2	Micos10	0.77	*P* < 0.05	1.20	*P* > 0.05
L-fucose kinase	Q7TMC8	Fcsk	0.70	*P* < 0.05	0.97	*P* > 0.05
Small integral membrane protein 12	Q78RX3	Smim12	0.68	*P* < 0.05	0.85	*P* > 0.05
T-lymphoma invasion and metastasis-inducing protein 2	Q6ZPF3	Tiam2	1.33	*P* < 0.05	1.07	*P* > 0.05
Diacylglycerol lipase-alpha	Q6WQJ1	Dagla	0.78	*P* < 0.05	1.14	*P* > 0.05
WASH complex subunit 2	Q6PGL7	Washc2	1.50	*P* < 0.05	0.96	*P* > 0.05
Palmitoyl-protein thioesterase ABHD10, mitochondrial	Q6PE15	Abhd10	0.81	*P* < 0.05	0.83	*P* > 0.05
Myogenesis-regulating glycosidase	Q69ZQ1	Myorg	1.27	*P* < 0.05	1.00	*P* > 0.05
Serine/threonine-protein kinase BRSK2	Q69Z98	Brsk2	1.25	*P* < 0.05	0.89	*P* > 0.05
Apoptosis-stimulating of p53 protein 1	Q62415	Ppp1r13b	0.76	*P* < 0.05	0.98	*P* > 0.05
Macrophage mannose receptor 1	Q61830	Mrc1	0.73	*P* < 0.05	0.81	*P* > 0.05
Zinc finger protein 638	Q61464	Znf638	1.21	*P* < 0.05	0.92	*P* > 0.05
Immunoglobulin-binding protein 1	Q61249	Igbp1	1.23	*P* < 0.05	0.94	*P* > 0.05
Dual specificity tyrosine-phosphorylation-regulated kinase 1A	Q61214	Dyrk1a	1.26	*P* < 0.05	1.06	*P* > 0.05
ELAV-like protein 2	Q60899	Elavl2	2.20	*P* < 0.05	1.20	*P* > 0.05
Long-chain fatty acid transport protein 1	Q60714	Slc27a1	0.80	*P* < 0.05	1.06	*P* > 0.05
Deoxynucleoside triphosphate triphosphohydrolase SAMHD1	Q60710	Samhd1	1.71	*P* < 0.05	1.01	*P* > 0.05
Laminin subunit alpha-2	Q60675	Lama2	0.67	*P* < 0.05	0.98	*P* > 0.05
Corticotropin-releasing factor-binding protein	Q60571	Crhbp	0.77	*P* < 0.05	0.95	*P* > 0.05
Protein jagunal homolog 1	Q5XKN4	Jagn1	0.66	*P* < 0.05	0.91	*P* > 0.05
WD repeat-containing protein 81	Q5ND34	Wdr81	0.76	*P* < 0.05	0.97	*P* > 0.05
Extracellular serine/threonine protein kinase FAM20C	Q5MJS3	Fam20c	1.25	*P* < 0.05	0.97	*P* > 0.05
Epimerase family protein SDR39U1	Q5M8N4	Sdr39u1	0.71	*P* < 0.05	1.05	*P* > 0.05
Volume-regulated anion channel subunit LRRC8B	Q5DU41	Lrrc8b	1.35	*P* < 0.05	0.94	*P* > 0.05
Optic atrophy 3 protein homolog	Q505D7	Opa3	0.71	*P* < 0.05	0.96	*P* > 0.05
Testis-expressed protein 10	Q3URQ0	Tex10	1.60	*P* < 0.05	0.95	*P* > 0.05
G protein-regulated inducer of neurite outgrowth 1	Q3UNH4	Gprin1	1.23	*P* < 0.05	1.02	*P* > 0.05
Glucose-fructose oxidoreductase domain-containing protein 1	Q3UHD2	Gfod1	0.81	*P* < 0.05	1.09	*P* > 0.05
Type 1 phosphatidylinositol 4,5-bisphosphate 4-phosphatase	Q3TWL2	Pip4p1	0.74	*P* < 0.05	0.92	*P* > 0.05
Keratin, type II cytoskeletal 2 epidermal	Q3TTY5	Krt2	0.76	*P* < 0.05	0.83	*P* > 0.05
Patatin-like phospholipase domain-containing protein 6	Q3TRM4	Pnpla6	0.74	*P* < 0.05	0.97	*P* > 0.05
Coiled-coil domain-containing protein 127	Q3TC33	Ccdc127	0.72	*P* < 0.05	1.13	*P* > 0.05
Mitogen-activated protein kinase kinase kinase 13	Q1HKZ5	Map3k13	0.69	*P* < 0.05	0.98	*P* > 0.05
Insulin-like growth factor-binding protein 5	Q07079	Igfbp5	1.22	*P* < 0.05	0.86	*P* > 0.05
Glutamate receptor ionotropic, NMDA 2D	Q03391	Grin2d	0.62	*P* < 0.05	0.94	*P* > 0.05
Junction plakoglobin	Q02257	Jup	0.82	*P* < 0.05	0.93	*P* > 0.05
Peroxiredoxin-5, mitochondrial	P99029	Prdx5	1.22	*P* < 0.05	0.93	*P* > 0.05
Amyloid-beta A4 precursor protein-binding family A member 2	P98084	Apba2	1.34	*P* < 0.05	0.91	*P* > 0.05
Adenylate cyclase type 8	P97490	Adcy8	0.60	*P* < 0.05	0.89	*P* > 0.05
Eukaryotic translation initiation factor 4E-binding protein 2	P70445	Eif4ebp2	1.21	*P* < 0.05	1.02	*P* > 0.05
Pituitary adenylate cyclase-activating polypeptide type I receptor	P70205	Adcyap1r1	1.52	*P* < 0.05	1.01	*P* > 0.05
Ran-binding protein 9	P69566	Ranbp9	0.79	*P* < 0.05	0.93	*P* > 0.05
SUMO-conjugating enzyme UBC9	P63280	Ube2i	0.76	*P* < 0.05	0.93	*P* > 0.05
Mitochondrial import inner membrane translocase subunit Tim13	P62075	Timm13	0.72	*P* < 0.05	0.98	*P* > 0.05
AP-1 complex subunit sigma-1A	P61967	Ap1s1	0.80	*P* < 0.05	0.91	*P* > 0.05
Nuclear protein localization protein 4 homolog	P60670	Nploc4	1.37	*P* < 0.05	1.11	*P* > 0.05
Ubiquitin carboxyl-terminal hydrolase 25	P57080	Usp25	0.68	*P* < 0.05	0.92	*P* > 0.05
Wolframin	P56695	Wfs1	0.68	*P* < 0.05	1.08	*P* > 0.05
Protoporphyrinogen oxidase	P51175	Ppox	0.81	*P* < 0.05	0.82	*P* > 0.05
Calpastatin	P51125	Cast	1.24	*P* < 0.05	1.09	*P* > 0.05
Arylsulfatase A	P50428	Arsa	0.77	*P* < 0.05	0.97	*P* > 0.05
Hematopoietic lineage cell-specific protein	P49710	Hcls1	0.77	*P* < 0.05	1.02	*P* > 0.05
Signal transducer and activator of transcription 3	P42227	Stat3	0.79	*P* < 0.05	0.97	*P* > 0.05
Radixin	P26043	Rdx	1.33	*P* < 0.05	1.10	*P* > 0.05
Lysosomal acid phosphatase	P24638	Acp2	0.78	*P* < 0.05	0.91	*P* > 0.05
Cytochrome P450 2D11	P24457	Cyp2d11	1.93	*P* < 0.05	1.09	*P* > 0.05
Microtubule-associated protein 2	P20357	Map2	1.22	*P* < 0.05	1.06	*P* > 0.05
Secretogranin-1	P16014	Chgb	1.38	*P* < 0.05	1.02	*P* > 0.05
Dystrophin	P11531	Dmd	0.79	*P* < 0.05	0.82	*P* > 0.05
Complement factor H	P06909	Cfh	1.36	*P* < 0.05	1.14	*P* > 0.05
Major prion protein	P04925	Prnp	0.76	*P* < 0.05	1.00	*P* > 0.05
Serine/threonine-protein kinase A-Raf	P04627	Araf	0.75	*P* < 0.05	1.00	*P* > 0.05
Keratin, type II cytoskeletal 1	P04104	Krt1	0.73	*P* < 0.05	1.17	*P* > 0.05
NADH-ubiquinone oxidoreductase chain 4	P03911	Mtnd4	0.78	*P* < 0.05	0.94	*P* > 0.05
Afamin	O89020	Afm	1.26	*P* < 0.05	0.81	*P* > 0.05
Sortilin-related receptor	O88307	Sorl1	0.68	*P* < 0.05	0.80	*P* > 0.05
Protein Wiz	O88286	Wiz	1.27	*P* < 0.05	1.03	*P* > 0.05
Protein tyrosine phosphatase type IVA 2	O70274	Ptp4a2	0.73	*P* < 0.05	0.95	*P* > 0.05
ATPase GET3	O54984	Get3	0.70	*P* < 0.05	0.98	*P* > 0.05
Caveolae-associated protein 1	O54724	Cavin1	0.78	*P* < 0.05	0.86	*P* > 0.05
Sialidase-1	O35657	Neu1	1.35	*P* < 0.05	1.06	*P* > 0.05
Glutamate carboxypeptidase 2	O35409	Folh1	2.18	*P* < 0.05	1.06	*P* > 0.05
3-hydroxyacyl-CoA dehydrogenase type-2	O08756	Hsd17b10	0.78	*P* < 0.05	0.90	*P* > 0.05
DNA-directed RNA polymerase II subunit RPB11	O08740	Polr2j	1.65	*P* < 0.05	0.94	*P* > 0.05
Phosphatidylinositol 3,4,5-trisphosphate 3-phosphatase and dual-specificity protein phosphatase PTEN	O08586	Pten	1.30	*P* < 0.05	1.00	*P* > 0.05
Rab11 family-interacting protein 2	G3XA57	Rab11fip2	0.69	*P* < 0.05	1.03	*P* > 0.05
Glutamate-rich protein 6	D3Z6S9	Erich6	0.76	*P* < 0.05	1.15	*P* > 0.05
Ryanodine receptor 3	A2AGL3	Ryr3	0.66	*P* < 0.05	0.86	*P* > 0.05

**Table 3:** List of the several sevoflurane-related significantly differential proteins in condition 3.

**Protein names**	**Proteins IDs**	**Gene name**	**P6S/6C**	***P***	**P60S/P6S**	***P***
Retinaldehyde-binding protein 1	Q9Z275	Rlbp1	0.61	*P* < 0.05	0.49	*P* < 0.05
Phospholemman	Q9Z239	Fxyd1	0.68	*P* < 0.05	4.96	*P* < 0.05
NPC intracellular cholesterol transporter 2	Q9Z0J0	Npc2	1.22	*P* < 0.05	0.78	*P* < 0.05
Histone-arginine methyltransferase CARM1	Q9WVG6	Carm1	0.83	*P* < 0.05	0.75	*P* < 0.05
RanBP-type and C3HC4-type zinc finger-containing protein 1	Q9WUB0	Rbck1	1.30	*P* < 0.05	1.25	*P* < 0.05
Prefoldin subunit 5	Q9WU28	Pfdn5	0.72	*P* < 0.05	0.61	*P* < 0.05
V-type proton ATPase subunit G 2	Q9WTT4	Atp6v1g2	1.41	*P* < 0.05	2.48	*P* < 0.05
Mitochondrial import inner membrane translocase subunit Tim23	Q9WTQ8	Timm23	0.78	*P* < 0.05	0.71	*P* < 0.05
A-kinase anchor protein 12	Q9WTQ5	Akap12	1.34	*P* < 0.05	0.70	*P* < 0.05
V-type proton ATPase subunit S1	Q9R1Q9	Atp6ap1	0.67	*P* < 0.05	0.61	*P* < 0.05
Diacylglycerol kinase epsilon	Q9R1C6	Dgke	0.82	*P* < 0.05	1.21	*P* < 0.05
Synaptotagmin-11	Q9R0N3	Syt11	0.82	*P* < 0.05	0.69	*P* < 0.05
Zinc finger E-box-binding homeobox 2	Q9R0G7	Zeb2	0.80	*P* < 0.05	0.72	*P* < 0.05
Serine racemase	Q9QZX7	Srr	0.66	*P* < 0.05	1.64	*P* < 0.05
DnaJ homolog subfamily A member 2	Q9QYJ0	Dnaja2	0.78	*P* < 0.05	0.75	*P* < 0.05
Activating signal cointegrator 1	Q9QXN3	Trip4	0.82	*P* < 0.05	0.80	*P* < 0.05
Alpha-N-acetylgalactosaminidase	Q9QWR8	Naga	0.71	*P* < 0.05	0.49	*P* < 0.05
Tubulin alpha-8 chain	Q9JJZ2	Tuba8	0.58	*P* < 0.05	2.02	*P* < 0.05
Phosphorylated adapter RNA export protein	Q9JJT9	Phax	0.82	*P* < 0.05	0.71	*P* < 0.05
Ribosomal oxygenase 1	Q9JJF3	Riox1	0.81	*P* < 0.05	0.79	*P* < 0.05
Transcription and mRNA export factor ENY2	Q9JIX0	Eny2	1.32	*P* < 0.05	0.69	*P* < 0.05
Solute carrier family 12 member 4	Q9JIS8	Slc12a4	1.26	*P* < 0.05	1.21	*P* < 0.05
Protein arginine N-methyltransferase 1	Q9JIF0	Prmt1	0.82	*P* < 0.05	0.60	*P* < 0.05
Palmdelphin	Q9JHU2	Palmd	0.55	*P* < 0.05	0.71	*P* < 0.05
Acidic leucine-rich nuclear phosphoprotein 32 family member B	Q9EST5	Anp32b	0.81	*P* < 0.05	0.63	*P* < 0.05
Tuftelin-interacting protein 11	Q9ERA6	Tfip11	0.77	*P* < 0.05	0.65	*P* < 0.05
Fructosamine-3-kinase	Q9ER35	Fn3k	0.83	*P* < 0.05	1.88	*P* < 0.05
Regulating synaptic membrane exocytosis protein 2	Q9EQZ7	Rims2	1.30	*P* < 0.05	1.47	*P* < 0.05
39S ribosomal protein L46, mitochondrial	Q9EQI8	Mrpl46	0.67	*P* < 0.05	0.53	*P* < 0.05
NADH dehydrogenase [ubiquinone] iron-sulfur protein 3, mitochondrial	Q9DCT2	Ndufs3	0.83	*P* < 0.05	1.48	*P* < 0.05
Methyltransferase-like 26	Q9DCS2	Mettl26	0.69	*P* < 0.05	2.42	*P* < 0.05
Eukaryotic translation initiation factor 3 subunit F	Q9DCH4	Eif3f	0.74	*P* < 0.05	0.82	*P* < 0.05
28S ribosomal protein S11, mitochondrial	Q9DCA2	Mrps11	0.81	*P* < 0.05	0.68	*P* < 0.05
NADH dehydrogenase [ubiquinone] 1 alpha subcomplex subunit 9, mitochondrial	Q9DC69	Ndufa9	0.83	*P* < 0.05	1.47	*P* < 0.05
Cytochrome P450 2S1	Q9DBX6	Cyp2s1	0.74	*P* < 0.05	0.80	*P* < 0.05
Protein phosphatase 1 regulatory subunit 12A	Q9DBR7	Ppp1r12a	1.29	*P* < 0.05	1.54	*P* < 0.05
Alpha-aminoadipic semialdehyde dehydrogenase	Q9DBF1	Aldh7a1	0.77	*P* < 0.05	1.31	*P* < 0.05
Cap-specific mRNA (nucleoside-2'-O-)-methyltransferase 1	Q9DBC3	Cmtr1	0.83	*P* < 0.05	0.80	*P* < 0.05
Calponin-3	Q9DAW9	Cnn3	1.33	*P* < 0.05	0.70	*P* < 0.05
Glycine amidinotransferase, mitochondrial	Q9D964	Gatm	0.80	*P* < 0.05	0.70	*P* < 0.05
Signal peptidase complex catalytic subunit SEC11C	Q9D8V7	Sec11c	0.83	*P* < 0.05	0.68	*P* < 0.05
DENN domain-containing protein 10	Q9D8N2	Dennd10	0.64	*P* < 0.05	0.40	*P* < 0.05
Splicing factor U2AF 35 kDa subunit	Q9D883	U2af1	0.73	*P* < 0.05	0.40	*P* < 0.05
EEF1A lysine methyltransferase 2	Q9D853	Eef1akmt2	1.33	*P* < 0.05	1.29	*P* < 0.05
Phospholysine phosphohistidine inorganic pyrophosphate phosphatase	Q9D7I5	Lhpp	0.68	*P* < 0.05	0.64	*P* < 0.05
Ribose-phosphate pyrophosphokinase 1	Q9D7G0	Prps1	0.78	*P* < 0.05	1.38	*P* < 0.05
Isobutyryl-CoA dehydrogenase, mitochondrial	Q9D7B6	Acad8	0.81	*P* < 0.05	1.30	*P* < 0.05
Synaptojanin-2-binding protein	Q9D6K5	Synj2bp	0.73	*P* < 0.05	1.73	*P* < 0.05
PHD finger protein 6	Q9D4J7	Phf6	0.75	*P* < 0.05	0.56	*P* < 0.05
Protein tweety homolog 1	Q9D3A9	Ttyh1	0.82	*P* < 0.05	1.69	*P* < 0.05
ADP-ribosylation factor-like protein 2-binding protein	Q9D385	Arl2bp	0.78	*P* < 0.05	0.51	*P* < 0.05
28S ribosomal protein S25, mitochondrial	Q9D125	Mrps25	0.67	*P* < 0.05	0.51	*P* < 0.05
5-methylcytosine rRNA methyltransferase NSUN4	Q9CZ57	Nsun4	0.81	*P* < 0.05	0.82	*P* < 0.05
Peroxiredoxin-like 2A	Q9CYH2	Prxl2a	0.75	*P* < 0.05	0.77	*P* < 0.05
Heterogeneous nuclear ribonucleoprotein A0	Q9CX86	Hnrnpa0	1.41	*P* < 0.05	0.71	*P* < 0.05
Peptidyl-prolyl cis-trans isomerase NIMA-interacting 4	Q9CWW6	Pin4	0.76	*P* < 0.05	0.51	*P* < 0.05
Mitochondrial fission process protein 1	Q9CRB8	Mtfp1	1.29	*P* < 0.05	1.69	*P* < 0.05
Methylsterol monooxygenase 1	Q9CRA4	Msmo1	0.64	*P* < 0.05	0.49	*P* < 0.05
Josephin-2	Q9CR30	Josd2	0.75	*P* < 0.05	1.52	*P* < 0.05
NADH dehydrogenase [ubiquinone] 1 alpha subcomplex subunit 6	Q9CQZ5	Ndufa6	1.28	*P* < 0.05	1.59	*P* < 0.05
Protein RER1	Q9CQU3	Rer1	0.70	*P* < 0.05	0.57	*P* < 0.05
Thioredoxin domain-containing protein 12	Q9CQU0	Txndc12	0.75	*P* < 0.05	0.56	*P* < 0.05
Protein transport protein Sec61 subunit beta	Q9CQS8	Sec61b	0.58	*P* < 0.05	0.38	*P* < 0.05
Solute carrier family 25 member 46	Q9CQS4	Slc25a46	0.81	*P* < 0.05	1.38	*P* < 0.05
Coactosin-like protein	Q9CQI6	Cotl1	0.80	*P* < 0.05	0.54	*P* < 0.05
CDGSH iron-sulfur domain-containing protein 2	Q9CQB5	Cisd2	0.81	*P* < 0.05	0.81	*P* < 0.05
39S ribosomal protein L49, mitochondrial	Q9CQ40	Mrpl49	0.79	*P* < 0.05	0.70	*P* < 0.05
EKC/KEOPS complex subunit Tp53rk	Q99PW4	Tp53rk	0.67	*P* < 0.05	0.33	*P* < 0.05
Long-chain-fatty-acid–CoA ligase ACSBG1	Q99PU5	Acsbg1	1.22	*P* < 0.05	1.32	*P* < 0.05
Tripartite motif-containing protein 12A	Q99PQ1	Trim12a	1.29	*P* < 0.05	0.70	*P* < 0.05
Acyl-CoA desaturase 3	Q99PL7	Scd3	0.71	*P* < 0.05	0.67	*P* < 0.05
RAF proto-oncogene serine/threonine-protein kinase	Q99N57	Raf1	1.43	*P* < 0.05	1.44	*P* < 0.05
BRCA1-associated protein	Q99MP8	Brap	0.82	*P* < 0.05	0.70	*P* < 0.05
Protein dpy-30 homolog	Q99LT0	Dpy30	0.76	*P* < 0.05	0.42	*P* < 0.05
Translation initiation factor eIF-2B subunit beta	Q99LD9	Eif2b2	0.64	*P* < 0.05	0.39	*P* < 0.05
ER membrane protein complex subunit 3	Q99KI3	Emc3	0.81	*P* < 0.05	0.73	*P* < 0.05
Ubiquitin carboxyl-terminal hydrolase 11	Q99K46	Usp11	1.31	*P* < 0.05	0.81	*P* < 0.05
Diphosphomevalonate decarboxylase	Q99JF5	Mvd	0.75	*P* < 0.05	0.33	*P* < 0.05
G-protein coupled receptor family C group 5 member B	Q923Z0	Gprc5b	1.26	*P* < 0.05	1.25	*P* < 0.05
Calcium/calmodulin-dependent protein kinase type II subunit gamma	Q923T9	Camk2g	0.83	*P* < 0.05	1.35	*P* < 0.05
tRNA modification GTPase GTPBP3, mitochondrial	Q923K4	Gtpbp3	1.57	*P* < 0.05	0.68	*P* < 0.05
Protein arginine N-methyltransferase 3	Q922H1	Prmt3	0.78	*P* < 0.05	1.33	*P* < 0.05
ADP-ribose glycohydrolase MACROD1	Q922B1	Macrod1	0.80	*P* < 0.05	1.31	*P* < 0.05
Gap junction gamma-3 protein	Q921C1	Gjc3	2.02	*P* < 0.05	2.61	*P* < 0.05
Vang-like protein 2	Q91ZD4	Vangl2	0.81	*P* < 0.05	0.64	*P* < 0.05
Egl nine homolog 1	Q91YE3	Egln1	0.82	*P* < 0.05	0.57	*P* < 0.05
ATP-dependent DNA helicase Q5	Q8VID5	Recql5	0.70	*P* < 0.05	0.42	*P* < 0.05
Voltage-dependent calcium channel gamma-8 subunit	Q8VHW2	Cacng8	1.21	*P* < 0.05	1.74	*P* < 0.05
Transcription initiation factor TFIID subunit 12	Q8VE65	Taf12	0.56	*P* < 0.05	0.30	*P* < 0.05
Ganglioside-induced differentiation-associated protein 1-like 1	Q8VE33	Gdap1l1	0.81	*P* < 0.05	0.73	*P* < 0.05
Purine-rich element-binding protein gamma	Q8R4E6	Purg	0.80	*P* < 0.05	0.78	*P* < 0.05
Heparan sulfate 2-O-sulfotransferase 1	Q8R3H7	Hs2st1	0.81	*P* < 0.05	0.58	*P* < 0.05
Protein C1orf43 homolog	Q8R092		0.75	*P* < 0.05	0.77	*P* < 0.05
Complement C1q tumor necrosis factor-related protein 4	Q8R066	C1qtnf4	0.75	*P* < 0.05	0.66	*P* < 0.05
Microtubule-associated protein RP/EB family member 2	Q8R001	Mapre2	0.83	*P* < 0.05	0.74	*P* < 0.05
Protein LZIC	Q8K3C3	Lzic	0.74	*P* < 0.05	0.43	*P* < 0.05
Very-long-chain (3R)-3-hydroxyacyl-CoA dehydratase 3	Q8K2C9	Hacd3	0.77	*P* < 0.05	0.69	*P* < 0.05
Membrane magnesium transporter 1	Q8K273	Mmgt1	0.77	*P* < 0.05	0.62	*P* < 0.05
COX assembly mitochondrial protein 2 homolog	Q8K199	Cmc2	0.81	*P* < 0.05	1.28	*P* < 0.05
Hydroxymethylglutaryl-CoA synthase, cytoplasmic	Q8JZK9	Hmgcs1	0.76	*P* < 0.05	0.39	*P* < 0.05
DNA polymerase theta	Q8CGS6	Polq	0.82	*P* < 0.05	1.83	*P* < 0.05
Guanine nucleotide-binding protein G(olf) subunit alpha	Q8CGK7	Gnal	1.55	*P* < 0.05	2.59	*P* < 0.05
Retinol dehydrogenase 13	Q8CEE7	Rdh13	0.60	*P* < 0.05	0.33	*P* < 0.05
Septin-10	Q8C650	Septin10	0.47	*P* < 0.05	0.22	*P* < 0.05
Calmodulin-regulated spectrin-associated protein 2	Q8C1B1	Camsap2	0.77	*P* < 0.05	0.74	*P* < 0.05
Rho-related GTP-binding protein RhoF	Q8BYP3	Rhof	0.73	*P* < 0.05	0.48	*P* < 0.05
Choline transporter-like protein 2	Q8BY89	Slc44a2	0.79	*P* < 0.05	1.41	*P* < 0.05
Ethanolamine-phosphate phospho-lyase	Q8BWU8	Etnppl	1.33	*P* < 0.05	1.80	*P* < 0.05
Gamma-secretase subunit APH-1A	Q8BVF7	Aph1a	0.79	*P* < 0.05	0.31	*P* < 0.05
Lipid droplet-associated hydrolase	Q8BVA5	Ldah	0.62	*P* < 0.05	0.48	*P* < 0.05
Ubiquitin carboxyl-terminal hydrolase 43	Q8BUM9	Usp43	0.82	*P* < 0.05	0.70	*P* < 0.05
Cilia- and flagella-associated protein 20	Q8BTU1	Cfap20	0.81	*P* < 0.05	0.54	*P* < 0.05
Inactive C-alpha-formylglycine-generating enzyme 2	Q8BPG6	Sumf2	0.82	*P* < 0.05	1.46	*P* < 0.05
Protein DPCD	Q8BPA8	Dpcd	0.67	*P* < 0.05	0.34	*P* < 0.05
Protein FRA10AC1 homolog	Q8BP78	Fra10ac1	0.81	*P* < 0.05	0.80	*P* < 0.05
Lysophosphatidic acid phosphatase type 6	Q8BP40	Acp6	1.55	*P* < 0.05	1.46	*P* < 0.05
Ubiquinone biosynthesis O-methyltransferase, mitochondrial	Q8BMS4	Coq3	0.75	*P* < 0.05	1.84	*P* < 0.05
Eukaryotic translation initiation factor 4E type 2	Q8BMB3	Eif4e2	1.43	*P* < 0.05	0.53	*P* < 0.05
Heat shock 70 kDa protein 13	Q8BM72	Hspa13	0.70	*P* < 0.05	0.64	*P* < 0.05
Serine/arginine-rich splicing factor 7	Q8BL97	Srsf7	1.21	*P* < 0.05	0.65	*P* < 0.05
Serine/threonine-protein kinase SMG1	Q8BKX6	Smg1	1.65	*P* < 0.05	0.56	*P* < 0.05
Pumilio homolog 3	Q8BKS9	Pum3	0.75	*P* < 0.05	0.69	*P* < 0.05
Zinc finger CCCH domain-containing protein 14	Q8BJ05	Zc3h14	0.18	*P* < 0.05	0.60	*P* < 0.05
TBC1 domain family member 10B	Q8BHL3	Tbc1d10b	1.21	*P* < 0.05	1.28	*P* < 0.05
Probable asparagine–tRNA ligase, mitochondrial	Q8BGV0	Nars2	0.70	*P* < 0.05	0.60	*P* < 0.05
NIPA-like protein 3	Q8BGN5	Nipal3	0.69	*P* < 0.05	2.19	*P* < 0.05
Glycerophosphocholine cholinephosphodiesterase ENPP6	Q8BGN3	Enpp6	1.77	*P* < 0.05	2.12	*P* < 0.05
Serine/threonine-protein phosphatase 2A 55 kDa regulatory subunit B gamma isoform	Q8BG02	Ppp2r2c	0.77	*P* < 0.05	0.65	*P* < 0.05
Beta-actin-like protein 2	Q8BFZ3	Actbl2	1.31	*P* < 0.05	0.69	*P* < 0.05
WD repeat-containing protein 82	Q8BFQ4	Wdr82	0.83	*P* < 0.05	0.66	*P* < 0.05
Mitofusin-1	Q811U4	Mfn1	0.82	*P* < 0.05	0.78	*P* < 0.05
U3 small nucleolar ribonucleoprotein protein MPP10	Q810V0	Mphosph10	0.73	*P* < 0.05	0.37	*P* < 0.05
28S ribosomal protein S10, mitochondrial	Q80ZK0	Mrps10	0.72	*P* < 0.05	0.76	*P* < 0.05
Sorting nexin-32	Q80ZJ7	Snx32	0.82	*P* < 0.05	0.80	*P* < 0.05
Myomegalin	Q80YT7	Pde4dip	0.81	*P* < 0.05	0.44	*P* < 0.05
Serine/threonine-protein phosphatase 1 regulatory subunit 10	Q80W00	Ppp1r10	0.59	*P* < 0.05	0.41	*P* < 0.05
Aldehyde dehydrogenase family 3 member B1	Q80VQ0	Aldh3b1	1.47	*P* < 0.05	1.79	*P* < 0.05
Tectonin beta-propeller repeat-containing protein 1	Q80VP0	Tecpr1	1.22	*P* < 0.05	1.31	*P* < 0.05
Sodium-dependent phosphate transporter 2	Q80UP8	Slc20a2	1.85	*P* < 0.05	1.52	*P* < 0.05
Ubiquitin-protein ligase E3C	Q80U95	Ube3c	0.83	*P* < 0.05	0.83	*P* < 0.05
Cullin-9	Q80TT8	Cul9	1.29	*P* < 0.05	0.73	*P* < 0.05
DnaJ homolog subfamily C member 16	Q80TN4	Dnajc16	0.75	*P* < 0.05	0.67	*P* < 0.05
Nischarin	Q80TM9	Nisch	1.21	*P* < 0.05	1.29	*P* < 0.05
Leucine-rich repeat and fibronectin type-III domain-containing protein 2	Q80TG9	Lrfn2	0.71	*P* < 0.05	0.72	*P* < 0.05
Synaptic vesicle membrane protein VAT-1 homolog-like	Q80TB8	Vat1l	1.22	*P* < 0.05	1.34	*P* < 0.05
CUB and sushi domain-containing protein 3	Q80T79	Csmd3	0.69	*P* < 0.05	0.36	*P* < 0.05
Pleckstrin homology domain-containing family A member 6	Q7TQG1	Plekha6	1.27	*P* < 0.05	1.35	*P* < 0.05
Tubulin polymerization-promoting protein	Q7TQD2	Tppp	1.24	*P* < 0.05	2.68	*P* < 0.05
Nucleosome assembly protein 1-like 4	Q78ZA7	Nap1l4	0.83	*P* < 0.05	0.66	*P* < 0.05
Purkinje cell protein 4-like protein 1	Q6W8Q3	Pcp4l1	1.20	*P* < 0.05	2.61	*P* < 0.05
F-box only protein 42	Q6PDJ6	Fbxo42	1.64	*P* < 0.05	1.61	*P* < 0.05
Armadillo-like helical domain-containing protein 3	Q6PD19	Armh3	0.81	*P* < 0.05	0.78	*P* < 0.05
Inositol hexakisphosphate kinase 1	Q6PD10	Ip6k1	0.77	*P* < 0.05	0.71	*P* < 0.05
Protein MTSS 2	Q6P9S0	Mtss2	1.31	*P* < 0.05	1.28	*P* < 0.05
FK506-binding protein 15	Q6P9Q6	Fkbp15	1.21	*P* < 0.05	0.75	*P* < 0.05
Structure-specific endonuclease subunit SLX4	Q6P1D7	Slx4	1.30	*P* < 0.05	0.41	*P* < 0.05
PILR alpha-associated neural protein	Q6P1B3	Pianp	1.51	*P* < 0.05	1.70	*P* < 0.05
Rho GTPase-activating protein 21	Q6DFV3	Arhgap21	0.82	*P* < 0.05	1.26	*P* < 0.05
SID1 transmembrane family member 1	Q6AXF6	Sidt1	1.31	*P* < 0.05	1.50	*P* < 0.05
Centrosomal protein of 170 kDa	Q6A065	Cep170	1.21	*P* < 0.05	0.74	*P* < 0.05
Pre-mRNA-splicing factor ISY1 homolog	Q69ZQ2	Isy1	0.80	*P* < 0.05	0.69	*P* < 0.05
COMM domain-containing protein 3	Q63829	Commd3	0.83	*P* < 0.05	0.80	*P* < 0.05
Tumor protein D52	Q62393	Tpd52	1.44	*P* < 0.05	1.33	*P* < 0.05
Replication protein A 32 kDa subunit	Q62193	Rpa2	0.71	*P* < 0.05	0.54	*P* < 0.05
Translocon-associated protein subunit delta	Q62186	Ssr4	1.35	*P* < 0.05	0.74	*P* < 0.05
Dystroglycan	Q62165	Dag1	0.83	*P* < 0.05	0.70	*P* < 0.05
Serum paraoxonase/arylesterase 2	Q62086	Pon2	0.49	*P* < 0.05	0.25	*P* < 0.05
28S ribosomal protein S31, mitochondrial	Q61733	Mrps31	0.74	*P* < 0.05	0.77	*P* < 0.05
Inter-alpha-trypsin inhibitor heavy chain H2	Q61703	Itih2	0.82	*P* < 0.05	0.77	*P* < 0.05
E3 ubiquitin/ISG15 ligase TRIM25	Q61510	Trim25	0.69	*P* < 0.05	0.68	*P* < 0.05
Protein phosphatase 1 regulatory subunit 1B	Q60829	Ppp1r1b	1.48	*P* < 0.05	2.61	*P* < 0.05
RAC-beta serine/threonine-protein kinase	Q60823	Akt2	0.65	*P* < 0.05	0.49	*P* < 0.05
Src substrate cortactin	Q60598	Cttn	1.31	*P* < 0.05	1.46	*P* < 0.05
G-protein coupled receptor-associated sorting protein 1	Q5U4C1	Gprasp1	1.25	*P* < 0.05	0.73	*P* < 0.05
DBF4-type zinc finger-containing protein 2 homolog	Q5SS00	Zdbf2	0.58	*P* < 0.05	0.22	*P* < 0.05
Echinoderm microtubule-associated protein-like 6	Q5SQM0	Eml6	0.72	*P* < 0.05	1.68	*P* < 0.05
RNA-binding protein 27	Q5SFM8	Rbm27	1.50	*P* < 0.05	0.76	*P* < 0.05
Capping protein inhibiting regulator of actin dynamics	Q5PR69	Crad	0.82	*P* < 0.05	0.51	*P* < 0.05
Neuralized-like protein 4	Q5NCX5	Neurl4	0.65	*P* < 0.05	0.54	*P* < 0.05
Sodium channel subunit beta-2	Q56A07	Scn2b	0.49	*P* < 0.05	4.21	*P* < 0.05
Capping protein, Arp2/3 and myosin-I linker protein 2	Q3V3V9	Carmil2	1.22	*P* < 0.05	1.76	*P* < 0.05
Transmembrane protein 237	Q3V0J1	Tmem237	0.60	*P* < 0.05	0.62	*P* < 0.05
Tau-tubulin kinase 2	Q3UVR3	Ttbk2	0.74	*P* < 0.05	0.67	*P* < 0.05
Protein FAM91A1	Q3UVG3	Fam91a1	0.77	*P* < 0.05	0.75	*P* < 0.05
Methyltransferase-like protein 17, mitochondrial	Q3U2U7	Mettl17	1.41	*P* < 0.05	1.45	*P* < 0.05
Ubiquitin-conjugating enzyme E2 variant 3	Q3U1V6	Uevld	0.74	*P* < 0.05	0.77	*P* < 0.05
UDP-N-acetylhexosamine pyrophosphorylase-like protein 1	Q3TW96	Uap1l1	1.25	*P* < 0.05	1.24	*P* < 0.05
NLR family member X1	Q3TL44	Nlrx1	0.76	*P* < 0.05	1.46	*P* < 0.05
Son of sevenless homolog 2	Q02384	Sos2	0.76	*P* < 0.05	0.43	*P* < 0.05
Nucleoside diphosphate kinase B	Q01768	Nme2	0.79	*P* < 0.05	0.52	*P* < 0.05
Proteasome subunit beta type-4	P99026	Psmb4	0.80	*P* < 0.05	0.82	*P* < 0.05
Phosphatidate cytidylyltransferase 1	P98191	Cds1	0.58	*P* < 0.05	0.50	*P* < 0.05
40S ribosomal protein S5	P97461	Rps5	0.83	*P* < 0.05	0.73	*P* < 0.05
Four and a half LIM domains protein 1	P97447	Fhl1	1.22	*P* < 0.05	1.22	*P* < 0.05
Lysosomal-trafficking regulator	P97412	Lyst	1.43	*P* < 0.05	1.36	*P* < 0.05
DNA replication licensing factor MCM2	P97310	Mcm2	0.53	*P* < 0.05	0.13	*P* < 0.05
Isochorismatase domain-containing protein 2A	P85094	Isoc2a	0.74	*P* < 0.05	1.90	*P* < 0.05
Syntaxin-4	P70452	Stx4	1.32	*P* < 0.05	1.23	*P* < 0.05
Ena/VASP-like protein	P70429	Evl	0.78	*P* < 0.05	0.71	*P* < 0.05
Plexin-A2	P70207	Plxna2	0.75	*P* < 0.05	0.60	*P* < 0.05
Dynein light chain 1, cytoplasmic	P63168	Dynll1	0.72	*P* < 0.05	0.64	*P* < 0.05
Thyroid hormone receptor alpha	P63058	Thra	0.76	*P* < 0.05	0.64	*P* < 0.05
Calmodulin regulator protein PCP4	P63054	Pcp4	1.29	*P* < 0.05	3.61	*P* < 0.05
Cellular retinoic acid-binding protein 1	P62965	Crabp1	1.34	*P* < 0.05	0.81	*P* < 0.05
60S ribosomal protein L32	P62911	Rpl32	0.81	*P* < 0.05	0.54	*P* < 0.05
60S ribosomal protein L30	P62889	Rpl30	0.78	*P* < 0.05	0.52	*P* < 0.05
60S ribosomal protein L23a	P62751	Rpl23a	0.81	*P* < 0.05	0.62	*P* < 0.05
Hippocalcin-like protein 1	P62748	Hpcal1	1.30	*P* < 0.05	0.71	*P* < 0.05
Ubiquitin-conjugating enzyme E2 H	P62257	Ube2h	0.61	*P* < 0.05	0.44	*P* < 0.05
40S ribosomal protein S15a	P62245	Rps15a	0.81	*P* < 0.05	0.64	*P* < 0.05
60S ribosomal protein L26	P61255	Rpl26	0.81	*P* < 0.05	0.61	*P* < 0.05
40S ribosomal protein S20	P60867	Rps20	0.76	*P* < 0.05	0.55	*P* < 0.05
Myocardin-related transcription factor B	P59759	Mrtfb	1.21	*P* < 0.05	1.42	*P* < 0.05
SH3 domain-binding protein 1	P55194	Sh3bp1	0.74	*P* < 0.05	1.69	*P* < 0.05
Tropomodulin-1	P49813	Tmod1	0.64	*P* < 0.05	2.65	*P* < 0.05
Glutamate decarboxylase 1	P48318	Gad1	0.73	*P* < 0.05	1.44	*P* < 0.05
60S ribosomal protein L13	P47963	Rpl13	0.82	*P* < 0.05	0.65	*P* < 0.05
60S ribosomal protein L6	P47911	Rpl6	0.83	*P* < 0.05	0.64	*P* < 0.05
Signal transducer and activator of transcription 5B	P42232	Stat5b	0.62	*P* < 0.05	0.50	*P* < 0.05
Tubulin–tyrosine ligase	P38585	Ttl	0.60	*P* < 0.05	0.65	*P* < 0.05
CD81 antigen	P35762	Cd81	0.58	*P* < 0.05	1.55	*P* < 0.05
Ras-related protein Rab-5C	P35278	Rab5c	0.78	*P* < 0.05	0.63	*P* < 0.05
cAMP-dependent protein kinase type II-beta regulatory subunit	P31324	Prkar2b	0.83	*P* < 0.05	0.80	*P* < 0.05
Progranulin	P28798	Grn	1.40	*P* < 0.05	1.36	*P* < 0.05
X-ray repair cross-complementing protein 5	P27641	Xrcc5	0.75	*P* < 0.05	0.75	*P* < 0.05
26S proteasome non-ATPase regulatory subunit 7	P26516	Psmd7	0.77	*P* < 0.05	0.72	*P* < 0.05
Splicing factor U2AF 65 kDa subunit	P26369	U2af2	1.79	*P* < 0.05	0.53	*P* < 0.05
Neuroendocrine convertase 2	P21661	Pcsk2	1.21	*P* < 0.05	1.25	*P* < 0.05
Neurofilament heavy polypeptide	P19246	Nefh	1.52	*P* < 0.05	2.05	*P* < 0.05
Complement C1q subcomponent subunit B	P14106	C1qb	1.20	*P* < 0.05	1.56	*P* < 0.05
Neuroendocrine protein 7B2	P12961	Scg5	1.22	*P* < 0.05	1.26	*P* < 0.05
Integrin beta-2	P11835	Itgb2	0.52	*P* < 0.05	3.11	*P* < 0.05
Cyclin-dependent kinase 1	P11440	Cdk1	0.81	*P* < 0.05	0.62	*P* < 0.05
Elongation factor 1-alpha 1	P10126	Eef1a1	0.79	*P* < 0.05	0.48	*P* < 0.05
Transmembrane protein 254c	P0DN91	Tmem254c	0.69	*P* < 0.05	0.38	*P* < 0.05
WAS/WASL-interacting protein family member 3	P0C7L0	Wipf3	1.27	*P* < 0.05	1.75	*P* < 0.05
Superoxide dismutase [Mn], mitochondrial	P09671	Sod2	0.74	*P* < 0.05	2.08	*P* < 0.05
Mast/stem cell growth factor receptor Kit	P05532	Kit	0.77	*P* < 0.05	2.37	*P* < 0.05
NADH-ubiquinone oxidoreductase chain 2	P03893	Mtnd2	1.47	*P* < 0.05	2.65	*P* < 0.05
Cytochrome c oxidase subunit 3	P00416	mt-Co3	1.32	*P* < 0.05	1.44	*P* < 0.05
3-keto-steroid reductase/17-beta-hydroxysteroid dehydrogenase 7	O88736	Hsd17b7	0.76	*P* < 0.05	0.56	*P* < 0.05
7-dehydrocholesterol reductase	O88455	Dhcr7	0.79	*P* < 0.05	0.46	*P* < 0.05
Metaxin-2	O88441	Mtx2	1.41	*P* < 0.05	1.60	*P* < 0.05
Electrogenic sodium bicarbonate cotransporter 1	O88343	Slc4a4	1.55	*P* < 0.05	1.74	*P* < 0.05
Transcription factor Sp3	O70494	Sp3	1.23	*P* < 0.05	0.70	*P* < 0.05
Homeobox protein PKNOX1	O70477	Pknox1	0.79	*P* < 0.05	1.38	*P* < 0.05
Tetraspanin-6	O70401	Tspan6	0.82	*P* < 0.05	0.63	*P* < 0.05
Stathmin-3	O70166	Stmn3	0.83	*P* < 0.05	0.59	*P* < 0.05
Transcription elongation factor SPT5	O55201	Supt5h	0.78	*P* < 0.05	0.69	*P* < 0.05
Barrier-to-autointegration factor	O54962	Banf1	0.76	*P* < 0.05	0.35	*P* < 0.05
Syndecan-4	O35988	Sdc4	1.70	*P* < 0.05	2.53	*P* < 0.05
Cleavage and polyadenylation specificity factor subunit 2	O35218	Cpsf2	0.83	*P* < 0.05	0.55	*P* < 0.05
Lysosomal alpha-mannosidase	O09159	Man2b1	0.82	*P* < 0.05	1.30	*P* < 0.05
Histone deacetylase 1	O09106	Hdac1	0.68	*P* < 0.05	0.63	*P* < 0.05
60 kDa SS-A/Ro ribonucleoprotein	O08848	RO60	0.77	*P* < 0.05	0.61	*P* < 0.05
YjeF N-terminal domain-containing protein 3	F6W8I0	Yjefn3	0.70	*P* < 0.05	2.54	*P* < 0.05
A-kinase anchor protein 5	D3YVF0	Akap5	1.23	*P* < 0.05	1.51	*P* < 0.05
3'-5' RNA helicase YTHDC2	B2RR83	Ythdc2	1.23	*P* < 0.05	1.22	*P* < 0.05
CDGSH iron-sulfur domain-containing protein 3, mitochondrial	B1AR13	Cisd3	1.27	*P* < 0.05	0.74	*P* < 0.05
Damage-control phosphatase ARMT1	A6H630	Armt1	0.79	*P* < 0.05	1.41	*P* < 0.05
Apical junction component 1 homolog	A2AJA9	Ajm1	0.75	*P* < 0.05	1.41	*P* < 0.05
Oxysterol-binding protein-related protein 9	A2A8Z1	Osbpl9	1.29	*P* < 0.05	0.80	*P* < 0.05

### 3.4. Gene ontology and Kyoto Encyclopedia of Genes and Genomes (KEGG) functional enrichment analysis of sevoflurane-related proteins

We used the DAVID database (https://david.ncifcrf.gov/home.jsp) for bioinformatics research, including GO and KEGG, to further determine the role of DEPs. The GO term is a collection of three primary ontologies: biological process (BP), molecular function (MF), and cellular component (CC), and GO functions of all DEPs have been annotated. We also performed a KEGG pathway analysis to identify the most important biochemical functions of identified DEPs.

**Figure 4:**
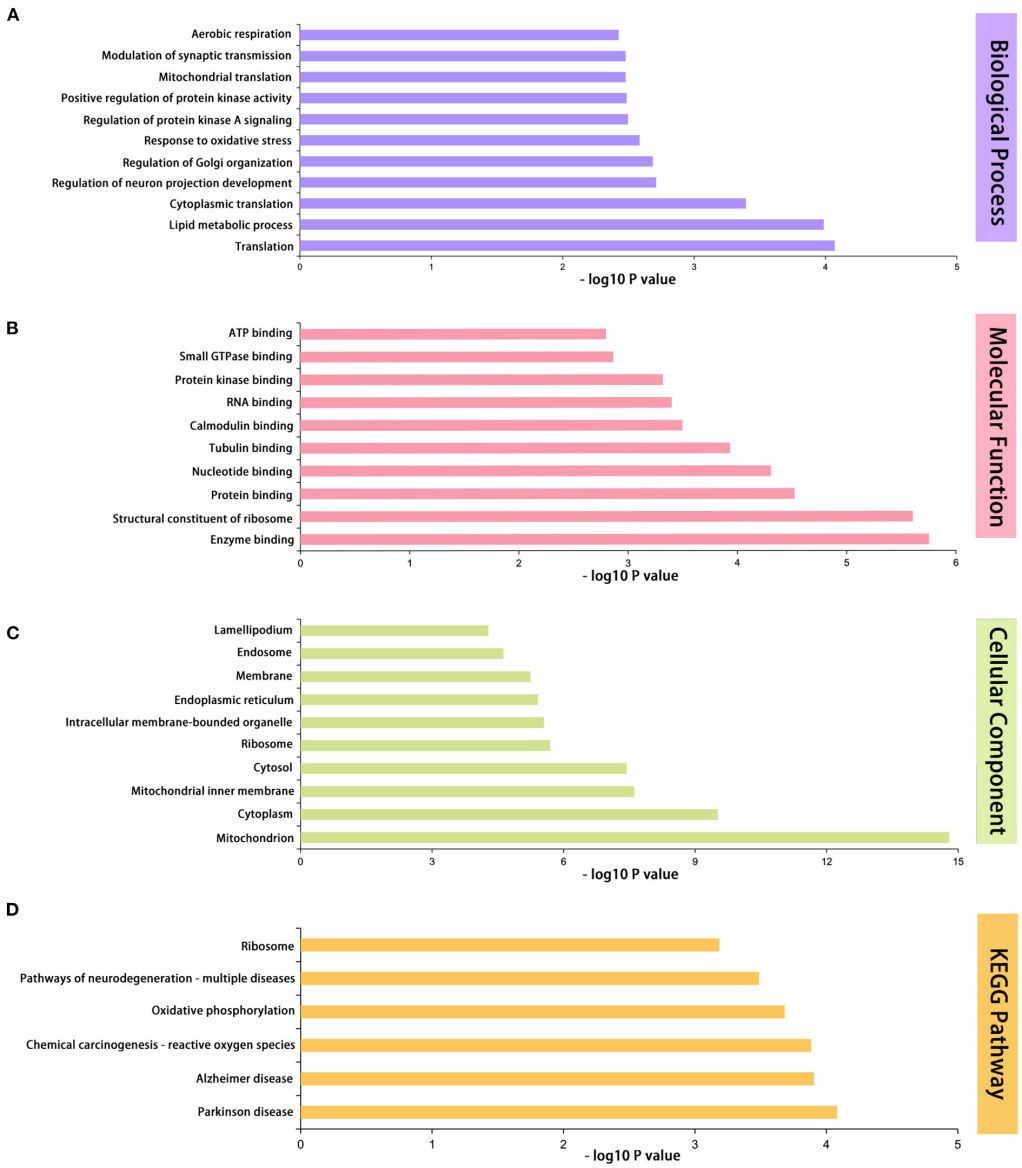
GO and KEGG functional enrichment analyses of 433 differentially expressed proteins. **(A)** The first 11 enriched GO terms of biological process. **(B)** The first 10 enriched GO terms of molecular functions. **(C)** The first 10 enriched GO terms of cellular components. **(D)** Differentially expressed protein pathway enriched by KEGG pathway analysis. *n* = 5 mice/group. GO, Gene Ontology; KEGG, Kyoto Encyclopedia of Genes and Genomes.

For BP terms, the first three terms were lipid metabolic process (7.2%), translation (4.3%), and response to oxidative (2.3%), according to the percentage. The top 11 significantly enriched BP terms were translation, lipid metabolic process, cytoplasmic translation, regulation of neuron projection development, regulation of Golgi organization, response to oxidative stress, regulation of protein kinase A signaling, positive regulation of protein kinase activity, mitochondrial translation, modulation of synaptic transmission, and aerobic respiration (Figure 4A).

For MF terms, protein binding (34.3%), nucleotide binding (13.9%), and RNA binding (8.3%) were ranked first. The top 10 significantly enriched MF terms were enzyme binding, structural constituent of ribosome, protein binding, nucleotide binding, tubulin binding, calmodulin binding, RNA binding, protein kinase binding, small GTPase binding, and ATP binding (Figure 4B).

For CC terms, we discovered that cytoplasm (47.6%), membrane (40.6%), and cytosol (29.7%) were the three most significant ratios. The top 10 CC terms that were significantly enriched were as follows: mitochondrion, cytoplasm, mitochondrial inner membrane, cytosol, ribosome, intracellular membrane-bounded organelle, endoplasmic reticulum, membrane, endosome, and lamellipodium (Figure 4C).

The top six enriched pathways with significant differences were as follows: Parkinson's disease, Alzheimer's disease, chemical carcinogenesis, reactive oxygen species (ROS), oxidative phosphorylation, pathways of neurodegeneration, multiple diseases, and ribosomes (Figure 4D).

### 3.5. Validation of CHGB, PTEN, MAP2c, and SOD2

**Figure 5:**
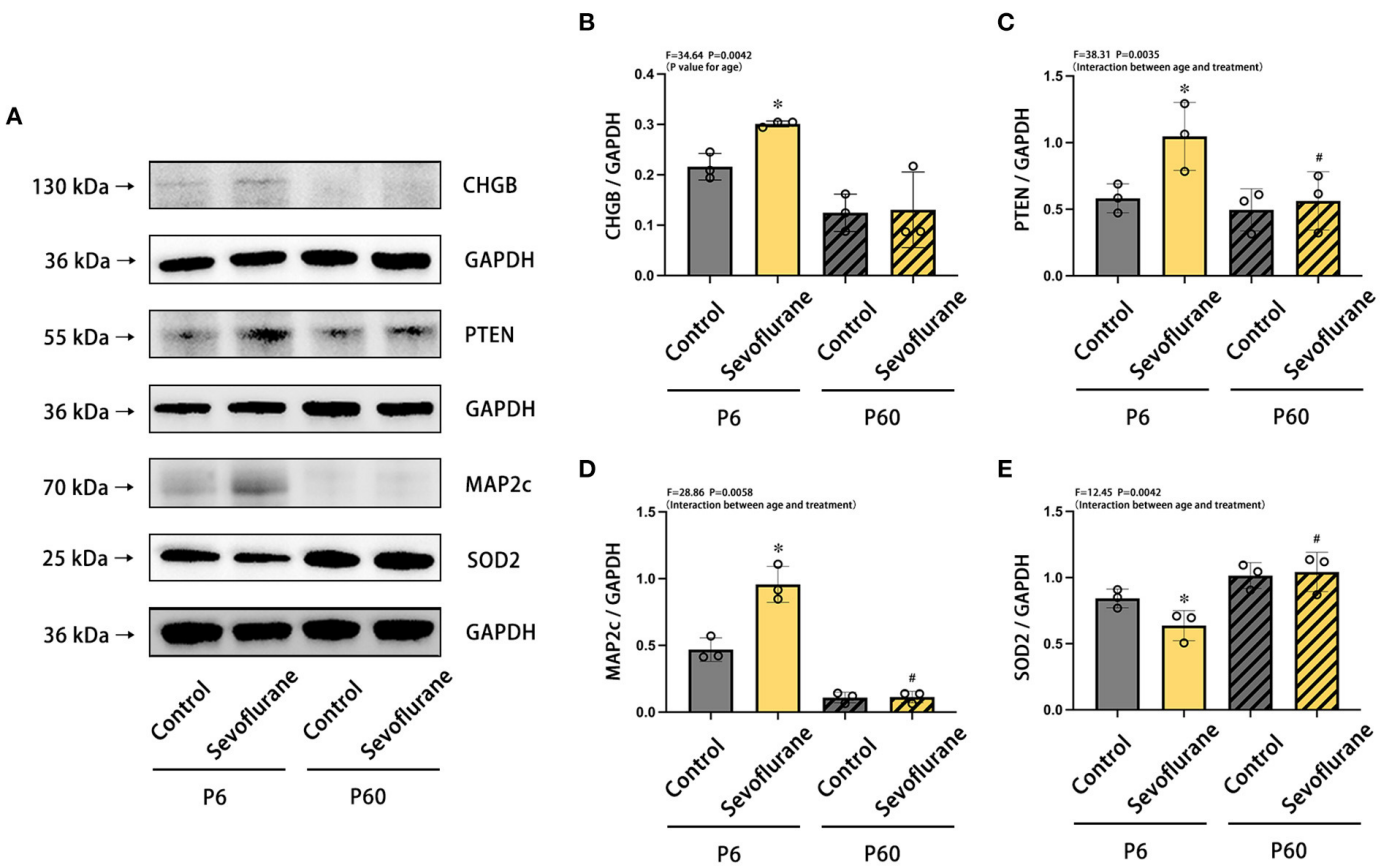
Differences in the expression of CHGB, PTEN, MAP2c, and SOD2 proteins in the cerebral cortex of neonatal and adult mice after multiple exposures to sevoflurane anesthesia. **(A)** Differences in the relative expression levels of **(B)** CHGB, **(C)** PTEN, **(D)** MAP2c, and **(E)** SOD2 in mice cerebral cortex at various ages and treatments. *n* = 5 mice/group. Results are expressed as mean ± standard deviation (SD). *P < 0.05 vs. P6 + control group, ^#^*P* < 0.05 vs. P6 + Sevoflurane group. P6, postnatal day 6.

We performed western blotting to validate the findings of the quantitative proteomics analysis (Figure 4). CHGB, PTEN, MAP2c, and SOD2 were selected based on their biological function (Supplementary Tables 1–4) and antibody availability. Compared with neonatal mice treated with oxygen, newborn mice subjected to multiple exposures of sevoflurane anesthesia exhibited elevated expression levels of CHGB, PTEN, and MAP2c protein in the cortex, whereas that of SOD2 was notably reduced (^*^P < 0.05, vs. P6 + control group). No statistically significant difference was detected between the adult groups (Figure 5).

## 4. Discussion

Based on previous research (7–11), we discovered that newborn mice exhibit neurotoxicity after multiple exposures to sevoflurane anesthesia. In the current study, we employed quantitative proteomic analysis using TMTpro(16-plek) tagging and LC-MS/MS to identify 443 DEPs. Moreover, we confirmed that these DEPs are related to unique mechanisms induced following multiple sevoflurane exposure-induced neurotoxicity during development. It is important to emphasize that we selected TMTpro as a label owing to the benefit of concurrent measurement across numerous samples, which markedly minimized batch effects (15).

The DAVID database was used to annotate and evaluate the functions and pathways of the DEPs. Herein, sevoflurane could induce neurotoxicity by influencing major mechanisms of mitochondrial energy metabolism (16), tau phosphorylation (8), and neuroinflammation (17). Our results corroborate those of previous reports, as determined using the GO/KEGG analysis. KEGG pathway analysis revealed that pathways of neurodegenerative diseases, including Parkinson's disease and Alzheimer's disease, were significantly enriched. Moreover, ROS- and oxidative phosphorylation-related pathways play a pivotal role in developmental neurotoxicity induced by multiple exposures to sevoflurane anesthesia. ROS is a toxic byproduct of aerobic metabolism and an indicator of oxidative stress-induced cellular damage. Typically, ROS are produced by mitochondria within the cell, and mitochondrial dysfunction elevates ROS levels to enhance inflammatory processes. Electron transport in the respiratory chain mediates oxidative phosphorylation. Sevoflurane inhibits the mitochondrial complex of the electron transport chain (18), facilitating the generation of oversized ROS. Using the GO analysis, we also identified mitochondrion-related enriched terms. These results indicate that the disrupted mitochondrial energy metabolism plays a significant role in sevoflurane-induced developmental neurotoxicity.

Considering the availability of antibodies and the biological functions of the protein (Supplementary Table 1), we selected four DEPs for verification: CHGB, PTEN, MAP2c, and SOD2. Herein, we noted that expression levels of CHGB, PTEN, and MAP2c increased, whereas those of SOD2 decreased. The results of the western blotting analysis were consistent with the trends predicted by proteome analysis, which demonstrated the reliability of TMT-based quantitative proteomics analysis.

CHGB is known to be associated with neurodegenerative diseases, including schizophrenia and Parkinson's disease (19, 20). As a type of neuroendocrine secretory granule protein, CHGB plays a key role in the regulated secretory pathway, impacting the secretion of trophic factors that modulate synaptic maturation of developing neurons (21), and is involved in the regulation of synaptic plasticity, which is related to memory formation (22). Combined with our findings exhibited in the BP of enriched GO terms (Figure 4A), multiple exposures to sevoflurane anesthesia could induce dysfunctional synaptic transmission in neonatal mice by increasing CHGB expression. PTEN protein is known to play a potential role in regulating the structure and plasticity of neurons, which hinders the Akt/mTOR signaling pathway to decrease the growth and proliferation of neurons and the activation of autophagy. In neonatal mice exposed to sevoflurane anesthesia, elevated PTEN expression may indicate neuronal loss in key brain regions that mediate cognitive function during neurodevelopment, and excessive activation of autophagy leads to autophagic programmed cell death (23, 24). The observed alterations in PTEN expression are consistent with several previous studies (25, 26). MAP2 exhibits microtubule stabilization activities that involve neurogenesis, morphogenesis, and migration for the development of axons and dendrites. MAP2 has three phenotypes: MAP2c (70 kDa), MAP2a, and MAP2b (both 280 kDa). MAP2a and MAP2b are expressed in the brains of adult mice but not newborn pups, and neonatal mice exhibit low MAP2b expression and abundant levels of MAP2c. MAP2c continues to decrease in abundance until adulthood (27). We selected MAP2c to verify quantitative proteomics owing to its specific expression during the newborn stage. Tau is expressed abundantly in neonatal mice when compared with that in adult mice, and some key phosphorylated-tau sites that detach from microtubules are increased in newborn mice exposed to sevoflurane anesthesia (8, 28). MAP2c and tau compete for binding sites, which is mediated by several factors (29). Therefore, under sevoflurane anesthesia, increased MAP2c brain expression may be influenced by elevated tau in neonatal mice, which could be a compensatory mechanism to maintain microtubule stability. SOD2, an enzyme belonging to the iron/manganese superoxide dismutase family and involved in the mitochondrial catabolic pathway, converts the superoxide anion to hydrogen peroxide, and the former is a potentially damaging product to the brain. The loss of SOD2 plays a critical role in the progression of neurodegenerative diseases (30, 31). Herein (32), the protein expression of SOD2 was attenuated in the hippocampus of neonatal mice exposed to 3% sevoflurane for 4 h. In addition, mitochondrion-related mechanisms are highly associated with sevoflurane-induced developmental neurotoxicity. Consequently, reduced SOD2 protein expression in newborn pups with multiple exposures to sevoflurane may result in elevated superoxide anion oxidation and oxidative stress damage in the brain.

In summary, we examined the mechanism underlying sevoflurane-induced neurotoxicity in newborn mice using TMT labeling and LS-MS/MS. Bioinformatic analysis was applied to identify DEPs, and we underlined the possibility of DNA damage through the mRNA surveillance pathway as a mechanism of sevoflurane-induced developmental neurotoxicity. Multiple sevoflurane exposures can cause brain damage and cognitive deficits in newborn mice, mediated *via* elevated levels of CHGB, PTEN, and MAP2c protein expression and reduced SOD2 expression. Our findings shed light on the mechanisms underlying the neurotoxicity induced by multiple exposures to sevoflurane anesthesia during development.

## Data availability statement

The original contributions presented in the study are publicly available. This data can be found at: ProteomeXchange, http://www.proteomexchange.org/, PXD037294.

## Ethics statement

All studies were approved by the Animal Experimental Ethics Committee at Tianjin Medical University General Hospital in Tianjin, China (Approval No. IRB2021-DWFL-210).

## Author contributions

JF, HL, and YZ performing all experiments, analyzing the data, and writing the original draft. YYa and XZ prepared the figures. YaYu conceived the study and reviewed the manuscript. YoYu supervised the study. All authors have read and approved the final version of the manuscript.

## Footnotes

^1^ https://www.uniprot.org/

## References

[1] Albayram O Herbert MK Kondo A Tsai CY Baxley S Lian X. Function and regulation of tau conformations in the development and treatment of traumatic brain injury and neurodegeneration. Cell Biosci. (2016) 6:59. 10.1186/s13578-016-0124-4 27980715 PMC5139118

[2] Sun M Xie Z Zhang J Leng Y. Mechanistic insight into sevoflurane-associated developmental neurotoxicity. Cell Biol Toxicol. (2021) 21:9677. 10.1007/s10565-021-09677-y 34766256 PMC9750936

[3] DiMaggio C Sun LS Kakavouli A Byrne MW Li G. A retrospective cohort study of the association of anesthesia and hernia repair surgery with behavioral and developmental disorders in young children. J Neurosurg Anesthesiol. (2009) 21:286–91. 10.1097/ANA.0b013e3181a71f11 19955889 PMC2789336

[4] Flick RP Katusic SK Colligan RC Wilder RT Voigt RG Olson MD. Cognitive and behavioral outcomes after early exposure to anesthesia and surgery. Pediatrics. (2011) 128:e1053–61. 10.1542/peds.2011-0351 21969289 PMC3307194

[5] Wilder RT Flick RP Sprung J Katusic SK Barbaresi WJ Mickelson C. Early exposure to anesthesia and learning disabilities in a population-based birth cohort. Anesthesiology. (2009) 110:796–804. 10.1097/01.anes.0000344728.34332.5d 19293700 PMC2729550

[6] McCann ME de Graaff JC Dorris L Disma N Withington D Bell G. Neurodevelopmental outcome at 5 years of age after general anaesthesia or awake-regional anaesthesia in infancy (GAS): an international, multicentre, randomised, controlled equivalence trial. Lancet. (2019) 393:664–77. 10.1016/S0140-6736(18)32485-1 30782342 PMC6500739

[7] Yu Y Yang M Zhuang X Pan J Zhao Y Yu Y. Effects of toxic apolipoprotein E fragments on Tau phosphorylation and cognitive impairment in neonatal mice under sevoflurane anesthesia. Brain Behav. (2022) 12:e2702. 10.1002/brb3.2702 35810473 PMC9392520

[8] Yu Y Yang Y Tan H Boukhali M Khatri A Yu Y. Tau contributes to sevoflurane-induced neurocognitive impairment in neonatal mice. Anesthesiology. (2020) 133:595–610. 10.1097/ALN.0000000000003452 32701572 PMC7429299

[9] Yang M Tan H Zhang K Lian N Yu Y Yu Y. Protective effects of Coenzyme Q10 against sevoflurane-induced cognitive impairment through regulating apolipoprotein E and phosphorylated Tau expression in young mice. Int J Dev Neurosci. (2020) 2020:jdn.10041. 10.1002/jdn.10041 32473608

[10] Li Y Zhang L Wang C Tang X Chen Y Wang X. Sevoflurane-induced learning deficits and spine loss via nectin-1/corticotrophin-releasing hormone receptor type 1 signaling. Brain Res. (2019) 1710:188–98. 10.1016/j.brainres.2018.12.010 30529655

[11] Yang Y Liang F Gao J Dong Y Zhang Y Yang G. Testosterone attenuates sevoflurane-induced tau phosphorylation and cognitive impairment in neonatal male mice. Br J Anaesth. (2021) 127:929–41. 10.1016/j.bja.2021.08.028 34686310 PMC9642834

[12] Tang W Dong M Teng F Cui J Zhu X Wang W. TMT-based quantitative proteomics reveals suppression of SLC3A2 and ATP1A3 expression contributes to the inhibitory role of acupuncture on airway inflammation in an OVA-induced mouse asthma model. Biomed Pharmacother. (2021) 134:111001. 10.1016/j.biopha.2020.111001 33341053

[13] Lian N Shen M Zhang K Pan J Jiang Y Yu Y. Drinking hydrogen-rich water alleviates chemotherapy-induced neuropathic pain through the regulation of gut microbiota. J Pain Res. (2021) 14:681–91. 10.2147/JPR.S288289 33732014 PMC7956896

[14] Park TJ Park JH Lee GS Lee JY Shin JH Kim MW. Quantitative proteomic analyses reveal that GPX4 downregulation during myocardial infarction contributes to ferroptosis in cardiomyocytes. Cell Death Dis. (2019) 10:835. 10.1038/s41419-019-2061-8 31685805 PMC6828761

[15] Li J Van Vranken JG Pontano Vaites L Schweppe DK Huttlin EL Etienne C. TMTpro reagents: a set of isobaric labeling mass tags enables simultaneous proteome-wide measurements across 16 samples. Nat Methods. (2020) 17:399–404. 10.1038/s41592-020-0781-4 32203386 PMC7302421

[16] Li M Guo J Wang H Li Y. Involvement of mitochondrial dynamics and mitophagy in sevoflurane-induced cell toxicity. Oxid Med Cell Longev. (2021) 2021:6685468. 10.1155/2021/6685468 33728028 PMC7937461

[17] Huang H Hu C Xu L Zhu X Zhao L Min J. The effects of hesperidin on neuronal apoptosis and cognitive impairment in the sevoflurane anesthetized rat are mediated through the PI3/Akt/PTEN and nuclear factor-kappaB (NF-kappaB) signaling pathways. Med Sci Monit. (2020) 26:e920522. 10.12659/MSM.920522 32296010 PMC7180331

[18] Hanley PJ Ray J Brandt U Daut J. Halothane, isoflurane and sevoflurane inhibit NADH:ubiquinone oxidoreductase (complex I) of cardiac mitochondria. J Physiol. (2002) 544:687–93. 10.1113/jphysiol.2002.025015 12411515 PMC2290615

[19] Shin JG Kim JH Park CS Kim BJ Kim JW Choi IG. Gender-specific associations between CHGB genetic variants and schizophrenia in a Korean population. Yonsei Med J. (2017) 58:619–25. 10.3349/ymj.2017.58.3.619 28332369 PMC5368149

[20] Wen G Pang H Wu X Jiang E Zhang X Zhan X. Proteomic characterization of secretory granules in dopaminergic neurons indicates chromogranin/secretogranin-mediated protein processing impairment in Parkinson's disease. Aging. (2021) 13:20335–58. 10.18632/aging.203415 34420933 PMC8436928

[21] Dominguez N van Weering JRT Borges R Toonen RFG Verhage M. Dense-core vesicle biogenesis and exocytosis in neurons lacking chromogranins A and B. J Neurochem. (2018) 144:241–54. 10.1111/jnc.14263 29178418 PMC5814729

[22] Tiwari NK Sathyanesan M Kumar V Newton SS. A comparative analysis of erythropoietin and carbamoylated erythropoietin proteome profiles. Life. (2021) 11:359. 10.3390/life11040359 33921564 PMC8073529

[23] Chen CY Chen J He L Stiles BL. PTEN: tumor suppressor and metabolic regulator. Front Endocrinol. (2018) 9:338. 10.3389/fendo.2018.00338 30038596 PMC6046409

[24] Xue H Xu Y Wang S Wu ZY Li XY Zhang YH. Sevoflurane post-conditioning alleviates neonatal rat hypoxic-ischemic cerebral injury *via* Ezh2-regulated autophagy. Drug Des Devel Ther. (2019) 13:1691–706. 10.2147/DDDT.S197325 31190748 PMC6528650

[25] Liu T Dong X Wang B Zhang S Bai J Ma W. Silencing of PTEN inhibits the oxidative stress damage and hippocampal cell apoptosis induced by Sevoflurane through activating MEK1/ERK signaling pathway in infant rats. Cell Cycle. (2020) 19:684–96. 10.1080/15384101.2020.1717041 32089060 PMC7145332

[26] Li X Wu Z Zhang Y Xu Y Han G Zhao P. Activation of autophagy contributes to sevoflurane-induced neurotoxicity in fetal rats. Front Mol Neurosci. (2017) 10:432. 10.3389/fnmol.2017.00432 29311820 PMC5744904

[27] Garner CC Matus A. Different forms of microtubule-associated protein 2 are encoded by separate mRNA transcripts. J Cell Biol. (1988) 106:779–83. 10.1083/jcb.106.3.779 3346325 PMC2115073

[28] Dong Y Liang F Huang L Fang F Yang G Tanzi RE. The anesthetic sevoflurane induces tau trafficking from neurons to microglia. Commun Biol. (2021) 4:560. 10.1038/s42003-021-02047-8 33980987 PMC8115254

[29] Sontag JM Nunbhakdi-Craig V White CL 3rd Halpain S Sontag E. The protein phosphatase PP2A/Balpha binds to the microtubule-associated proteins Tau and MAP2 at a motif also recognized by the kinase Fyn: implications for tauopathies. J Biol Chem. (2012) 287:14984–93. 10.1074/jbc.M111.338681 22403409 PMC3340226

[30] Fracassi A Marcatti M Zolochevska O Tabor N Woltjer R Moreno S. Oxidative damage and antioxidant response in frontal cortex of demented and nondemented individuals with Alzheimer's neuropathology. J Neurosci. (2021) 41:538–54. 10.1523/JNEUROSCI.0295-20.2020 33239403 PMC7821866

[31] Lin C-H Wei P-C Chen C-M Huang Y-T Lin J-L Lo Y-S. Lactulose and melibiose attenuate MPTP-induced Parkinson's disease in mice by inhibition of oxidative stress, reduction of neuroinflammation and up-regulation of autophagy. Front Aging Neurosci. (2020) 12:226. 10.3389/fnagi.2020.00226 32848705 PMC7396622

[32] Yang F Zhang Y Tang Z Shan Y Wu X Liu H. Hemin treatment protects neonatal rats from sevoflurane-induced neurotoxicity *via* the phosphoinositide 3-kinase/Akt pathway. Life Sci. (2020) 242:117151. 10.1016/j.lfs.2019.117151 31843526

