# Supplementary material for: Tandem mass tag-based quantitative proteomic analysis of effects of multiple sevoflurane exposures on the cerebral cortex of neonatal and adult mice
Source: Front Neurol. 2022 Dec 13;13:1056947. doi: 10.3389/fneur.2022.1056947 (PMC9792844; doi:10.3389/fneur.2022.1056947)
Supplement: Supplementary file 1 [file Data_Sheet_1.docx]

**Supplementary Table to: Frontiers in Neurology**

**Tandem Mass Tag-Based Quantitative Proteomic Analysis of Effects of Multiple Sevoflurane Exposures on the Cerebral Cortex of Neonatal and Adult Mice**

**TABLE S1**

**The list of the proteins related to the top eleven biological process terms of the GO.**

| **Biological process** | **Gene names of the related protein names** |
| --- | --- |
| Translation | Rps5, Mrpl16, Mrps11, Eif2b2, Eif3f, Mrpl49, Nars2, Eef1a1, Mrpl9, Eif4e2, Mettl17, Mrps10, Rpl23a, Rps15a, Rpl26, Rpl32, Rpl13, Rps20, Rpl6 |
| Lipid metabolic process | Ptgr2, Npc2, Pip4p1, Acsl5, Aldh3b1, Acsbg1, Osbpl10, Acad8, Hacd3, Hsd17b7, Hmgcs1, Naga, Plcxd3, Rdh13, Dhcr7, Dagla, Scd3, Enpp6, Mvd, Cyp2s1, Ephx1, Agpat3, Slc27a1, Pnpla6, Arsa, Hsd17b10, Pten, Neu1, Msmo1, Dgke, Cds1, Acp6 |
| Cytoplasmic translation | Rps5, Rpl23a, Rps15a, Rpl26, Rpl30, Rpl13, Rpl32, Rps20, Rpl6 |
| Regulation of neuron projection development | Slc39a12, Znf365, Ntng1, Pten, Brsk2, Camk2g |
| Regulation of Golgi organization | Rbsn, Armh3, Pde4dip, Camsap2 |
| Response to oxidative stress | Sod2, Prdx5, Als2, Prnp, Aldh3b1, Pon2, Ndufa6, Camk2g, Lrrk2, Usp25 |
| Regulation of protein kinase A signaling | Akap5, Akap12, Prkar2b, Lrrk2 |
| Positive regulation of protein kinase activity | Xrcc5, Map3k13, Als2, Dag1, Gprc5b, Lrrk2, Sdc4 |
| Mitochondrial translation | Mrpl16, Mrps10, Mrps31, Mrps25, Mrps11, Mrpl49, Mrpl9, Mrpl46 |
| Modulation of synaptic transmission | Ntng1, Lrfn2, Dgke, Akap12, Eif4ebp2, Adcy8, Stat3, Prkar2b |
| Aerobic respiration | Ndufa9, Mtnd4, mt-Co3, Ndufa6, Uqcr10, Ndufs3, mt-Nd2 |

**TABLE S2**

**The list of the proteins related to the top ten molecular function terms of the GO.**

| **Molecular function** | **Gene names of the related protein names** |
| --- | --- |
| Enzyme binding | Npc2, Slx4, Prmt1, Map3k13, Dynll1, Hdac1, Nme2, Hacd3, Ranbp9, Banf1, Sod2, Rpa2, Phf6, Tuba8, Aph1a, Ephx1, Igbp1, Mcm2, Armt1, Timm23, Raf1, Ube2i, Egln1, Pten, Supt5h, Pde4dip, Cotl1, Iqcb1, U2af2 |
| Structural constituent of ribosome | Rps5, Mrpl16, Mrps31, Mrps25, Mrps11, Mrpl49, Mrpl9, Mrps10, Rpl23a, Rps15a, Rpl26, Rpl30, Rpl32, Rpl13, Rps20, Rpl6, Mrpl46 |
| Protein binding | Dpcd, Mrc1, Srsf7, Pip4p1, Sp3, U2af1, Fam241b, Cdk1, Rbck1, Dynll1, Rpl23a, Nme2, Evl, Hacd3, Apbb1, Cavin1, Lrfn2, Cnst, Stat3, Grn, Xrcc5, Cttn, Prnp, Eef1a1, Cd81, Carmil2, Mrtfb, Prkar2b, Stx6, Thra, Iqcb1, Palmd, Synj2bp, Wdr43, Wiz, Pknox1, Taf12, Sec61b, Rps6ka2, Prmt3, Mfn1, Tab1, Itsn2, Sumf2, Fhl1, Eif4ebp2, Lrrk2, Prmt1, Adcyap1r1, Ppp1r12a, Cdk16, Plxna2, Srr, Brap, Mapre2, Ranbp9, Rpa2, Rab3b, Gprasp1, Dagla, Ap1s1, Ecsit, Ndufs3, Dnajb6, Atl1, Sorcs1, Dapk1, Itgb2, Araf, Sel1l, Tmem237, Optn, Cox7c, Pten, Polr2j, Neu1, Slc44a2, Carm1, Sorl1, Cdk5rap3, Akap5, Scyl3, Cotl1, Cpne1, Cnot9, U2af2, Trim25, Atp6v1g2, Ntng1, Rasgrf1, Akt2, Rpgrip1, Sos2, Ppp1r10, Riox1, Dag1, Syngr3, Osbpl1a, Jup, Als2, Apba2, Palm2, Map2, Kit, Abcf3, Wdr82, Gja1, Igbp1, Stmn3, Slc27a1, Stx4, Cfh, Rims2, Dmd, Tiam2, Pde4dip, Dyrk1a, Prdx5, Rdx, Eif3f, Myorg, Rab5c, Syt11, Carhsp1, Arl2bp, Atg16l1, Hdac1, Nlrx1, Sod2, Adam17, Crybb1, Rbfox3, Hcls1, Cep170, Adcy8, Sdc4, Aph1a, Rragc, Camsap2, Raf1, Ube2i, Tpd52, Brsk2, Ythdc2, Ndufa9, Chgb, Vangl2, Stat5b, Rpl26, Tfip11, Hpcal1, Slc4a4 |
| Nucleotide binding | Hspa13, Smg1, Acsl5, Akt2, Actbl2, Acsbg1, Vwa8, Cdk1, Nme2, Camk2g, Tuba8, Recql5, Kit, Abcf3, Xrcc5, Gtpbp3, Samhd1, Mcm2, Ddx47, Slc27a1, Eef1a1, Fn3k, Prkar2b, Dgke, Diras1, Snrk, Rps6ka2, Dyrk1a, Mfn1, Polq, Rab5c, Nars2, Ip6k1, Lrrk2, Srr, Cdk16, Map3k13, Nlrx1, Get3, Camk1d, Rab3b, Septin10, Gnal, Ube2h, Adcy8, Mvd, Atl1, Rragc, Dapk1, Fam20c, Araf, Ube2i, Raf1, Brsk2, Cul9, Rhof, Gfod1, Ythdc2, Ttl, Fcsk, Spag1, Prps1 |
| Tubulin binding | Dyrk1a, Gja1, Stmn3, Tppp, Prnp, Dag1, Map2, Lrrk2, Phf6 |
| Calmodulin binding | Dapk1, Wfs1, Camsap2, Eef1a1, Akap12, Ryr3, Camk1d, Pcp4, Akap5, Iqcb1, Cnn3, Camk2g, Adcy8, Map2 |
| RNA binding | Hnrnpa0, Rps5, Srsf7, RO60, Ppp1r10, Riox1, U2af1, Carhsp1, Rpl23a, Phax, Rbfox3, Cavin1, Nsun4, Rpl6, Xrcc5, Samhd1, Slirp, Elavl2, Ddx47, Rbm27, Khdrbs3, Ythdc2, Znf638, Eif4e2, Rbm14, Rpl26, Pcp4, Zc3h14, Cpne1, Rpl13, Rpl30, Pum3, Sidt1, Rps20, Cpsf2, Wdr43, U2af2 |
| Protein kinase binding | Hnrnpa0, Rasgrf1, Actbl2, Rab11fip2, Trim12a, Ppp1r12a, Nisch, Mapre2, Trip4, Jup, Hcls1, Map2, Stat3, mt-Nd2, Lyst, Itgb2, Brsk2, Eef1a1, Nefh, Rhof, Gprc5b, Prkar2b, Pten, Slc12a4, Cdk5rap3, Akap5 |
| Small GTPase binding | Rasgrf1, Rab11fip2, Raf1, Stx4, Optn, Rims2, Lrrk2, Atp6ap1, Adcyap1r1, Rab11fip5, Ranbp9, Rbsn, Sorl1, Als2, Dennd10, Mtss2 |
| ATP binding | Hspa13, Smg1, Acsl5, Akt2, Eif2b2, Actbl2, Acsbg1, Vwa8, Cdk1, Nme2, Camk2g, Recql5, Kit, Abcf3, Xrcc5, Mcm2, Ddx47, Ttbk2, Fn3k, Dgke, Snrk, Rps6ka2, Dyrk1a, Polq, Nars2, Tp53rkb, Ip6k1, Lrrk2, Srr, Cdk16, Map3k13, Nlrx1, Get3, Camk1d, Dnaja2, Ube2h, Adcy8, Mvd, Dapk1, Fam20c, Araf, Ube2i, Raf1, Brsk2, Cul9, Ythdc2, Atp6ap1, Ttl, Fcsk, Scyl3, Prps1 |

**TABLE S3**

**The list of the proteins related to the top ten cellular component terms of the GO.**

| **Cellular component** | **Gene names of the related protein names** |
| --- | --- |
| Mitochondrion | Gatm, Hspa13, Mrps25, Macrod1, Acsl5, Akt2, Fam162a, Opa3, Abhd10, Pon2, Mrpl49, Vwa8, Cdk1, Dynll1, Mrps10, Prxl2a, Nme2, Acad8, Sdhc, Rdh13, Mtfp1, Timm13, Cavin1, Nsun4, Cmc2, Uqcr10, Brinp3, Grn, Stat3, Eny2, Gtpbp3, Gja1, Mrps11, Slirp, Mtnd4, Slc27a1, Timm23, Hsd17b10, Yjefn3, Cfh, Thra, Rps15a, Cisd3, Dmac2l, mt-Co3, Gad1, Mtx2, Acp6, Synj2bp, Coq3, Mrpl46, Ptgr2, Mfn1, Mrps31, Psmb4, Prdx5, Nars2, Aldh7a1, Slc25a46, Lrrk2, Arl2bp, Rab11fip5, Cisd2, Nlrx1, Sod2, Tppp, Cyp2d11, Micos10, Hcls1, Wdr81, Ecsit, Ndufs3, mt-Nd2, Aph1a, Mrpl16, Ppox, Araf, Raf1, Ndufa6, Nefh, Mrpl9, Isoc2a, Mettl17, Cox7c, Fem1aa, Triap1, Pten, Ndufa9, Tex10, Slc44a2, Etnppl |
| Cytoplasm | Rps5, Smg1, Ccdc50, Srsf7, Trim12a, Cdk1, Neurl4, Evl, Plcxd3, Apbb1, Rpl32, Nsun4, Tmod1, Gtpbp3, Cttn, Ppp1r13b, Prnp, Pfdn5, Eef1a1, Carmil2, Cmtr1, Akap12, Mrtfb, Rps15a, Iqcb1, Mtx2, Rps20, Mtss2, Pknox1, Rps6ka2, Prmt3, Itsn2, Aldh3b1, Fhl1, Usp11, Cast, Eif4ebp2, Lrrk2, Adcyap1r1, Ppp1r12a, Rab11fip5, Cdk16, Map3k13, Nisch, Hmgcs1, Mapre2, Ranbp9, Rab3b, Gprasp1, Dagla, Trappc2l, Fkbp15, Dapk1, Rbm27, Rhof, Optn, Josd2, Fem1aa, Pten, Carm1, Rbm14, Spag1, Akap5, Scyl3, Rpl13, Rlbp1, Akt2, Acsbg1, Vwa8, Prxl2a, Apba2, Ppp1r1b, Cyp2s1, Igbp1, Gja1, Mcm2, Slirp, Pogz, Slc27a1, Ttbk2, Enoph1, Cfh, Tiam2, Acp6, Ptgr2, Dyrk1a, Psmb4, Rdx, Prdx5, Eif3f, Aldh7a1, Carhsp1, Arl2bp, Phax, Get3, Rbfox3, Dnaja2, Hcls1, Aasdhppt, Mvd, Nap1l4, Rragc, Lyst, Camsap2, Brsk2, Anp32b, Ythdc2, Clcc1, Rpl26, Tfip11, Commd5, Osbpl10, Dynll1, Rpl23a, Nme2, Banf1, Cavin1, Rpl6, Camk2g, Tuba8, Stat3, Xrcc5, Afm, Arsa, Prkar2b, Eml6, Eif4e2, Thra, Pcp4, Rpl30, Dgke, Gad1, Palmd, Pin4, Snrk, Ubqln4, Copb1, RO60, Tp53rkb, Dcun1d1, Prmt1, Srr, Brap, Naga, Cyp2d11, Ecsit, Dnajb6, Atl1, Eef1akmt2, Cul9, Isoc2a, Neu1, Tex10, Kcnip2, Arhgap21, Cdk5rap3, Cotl1, Cpne1, Usp25, Cnot9, Trim25, Hspa13, Rasgrf1, Eif2b2, Actbl2, Dag1, Lrrc8b, Trip4, Jup, Als2, Mmgt1, Sh3bp1, Map2, Recql5, Kit, Commd10, Stmn3, Stx4, Egln1, Dmd, Dmac2l, Pde4dip, Crabp1, Zc3h14, Commd3, Cfap20, Lhpp, Syt11, Ptp4a2, Ip6k1, Atg16l1, Hdac1, Sod2, Camk1d, Adam17, Nploc4, Tppp, Tbc1d10b, Septin10, Cep170, Wdr81, Wipf3, Znf365, Fam20c, Raf1, Ube2i, Tpd52, Nefh, Stat5b, Armh3, Prps1 |
| Mitochondrial inner membrane | Gatm, Mfn1, Mrps25, Mrps31, Acsl5, Mrpl49, Lrrk2, Mrps10, Sod2, Sdhc, Timm13, Rdh13, Mtfp1, Micos10, Uqcr10, Stat3, Ndufs3, mt-Nd2, Mrpl16, Mrps11, Ppox, Mtnd4, Slc27a1, Timm23, Ndufa6, Hsd17b10, Mrpl9, Cox7c, Ndufa9, Dmac2l, mt-Co3, Coq3, Mrpl46 |
| Cytosol | Cracd, Rps5, Ccdc50, Fam162a, Abhd10, Trim12a, Osbpl10, Sp3, Cdk1, Dynll1, Rpl23a, Banf1, Cavin1, Rpl32, Rpl6, Stat3, Cttn, Ppp1r13b, Pfdn5, Prnp, Eef1a1, Akap12, Stx6, Thra, Rps15a, Osbpl9, Ylpm1, Rpl30, Snx32, Dgke, Rps20, Prmt3, Polq, Ubqln4, Copb1, Tab1, RO60, Aldh3b1, Fhl1, Nars2, Usp11, Dcun1d1, Cast, Lrrk2, Prmt1, Ppp1r12a, Cdk16, Brap, Nisch, Hmgcs1, Ranbp9, Rab3b, Gprasp1, Ap1s1, Ecsit, Dnajb6, Eef1akmt2, Trappc2l, Dapk1, Araf, Cul9, Gprc5b, Optn, Josd2, Pten, Washc2, Carm1, Spag1, Rbsn, Cdk5rap3, Akap5, Cotl1, Rpl13, Cpne1, Rlbp1, Cnn3, Usp25, Trim25, Rasgrf1, Akt2, Osbpl1a, Ubxn4, Trip4, Jup, Als2, Sh3bp1, Recql5, Gja1, Igbp1, Stmn3, Pogz, Pnpla6, Ttbk2, Fn3k, Egln1, Crabp1, Usp12, Lhpp, Ptgr2, Psmb4, Prdx5, Rdx, Aldh7a1, Ip6k1, Carhsp1, Arl2bp, Slx4, Atg16l1, Hdac1, Adam17, Nploc4, Dnaja2, Tppp, Tbc1d10b, Hcls1, Cep170, Wdr81, Aasdhppt, Mvd, Ppp2r2c, Lyst, Camsap2, Raf1, Ube2i, Stat5b, Rpl26, Zeb2, Commd5, Armh3, Unc5a, Prps1 |
| Ribosome | Rps5, Mrpl16, Mrps31, Mrps25, Mrps11, Mrpl49, Mrpl9, Dmd, Mrps10, Rpl23a, Rps15a, Rpl30, Rpl32, Rpl13, Rps20, Rpl6, Mrpl46 |
| Intracellular membrane-bounded organelle | Hspa13, Akt2, Rab11fip2, Pon2, Acsbg1, Osbpl10, Dag1, Fam241b, Osbpl1a, Als2, Cavin1, Cnst, Dhcr7, Scd3, Cyp2s1, Cttn, Gja1, Slc27a1, Prnp, Stard3nl, Cmtr1, Cisd3, Osbpl9, Prdx5, Copb1, Lrrk2, Adcyap1r1, Rab11fip5, Cisd2, Nisch, Ryr3, Rab3b, Cyp2d11, Pcsk2, Ap1s1, Eef1akmt2, Znf365, Ephx1, Trappc2l, Rragc, Tecpr1, Gprc5b, Rhof, Znf638, Man2b1, Neu1, Washc2, Clcc1, Rbsn, Cdk5rap3 |
| Endoplasmic reticulum | Hspa13, Tmem33, Ldah, Acsl5, Acsbg1, Txndc12, Rer1, Lrrc8b, Jagn1, Ubxn4, Hacd3, Hsd17b7, Apbb1, Cavin1, Mmgt1, Dhcr7, Rpl6, Scd3, Brinp3, Grn, Cyp2s1, Gja1, Agpat3, Slc27a1, Prnp, Pnpla6, Arsa, Hsd17b10, Msmo1, Emc3, Pum3, Sec61b, Npc2, Ubqln4, Wfs1, Slc33a1, Sumf2, Ssr4, Myorg, Cast, Lrrk2, Cisd2, Ryr3, Get3, Nploc4, Cyp2d11, Aph1a, Atl1, Ephx1, Trappc2l, Sec11c, Tpd52, Brsk2, Sel1l, Ythdc2, Atp6ap1, Clcc1, Sorl1, Rpl13, Cds1, Usp25 |
| Membrane | Pianp, Mrc1, Cacng8, Tmem33, Fxyd1, Grin2d, Pip4p1, Fam162a, Adgra1, Rnf141, Rab11fip2, Pon2, Osbpl10, Fam241b, Rer1, Dynll1, Hacd3, Hsd17b7, Acp2, Timm13, Apbb1, Cavin1, Lrfn2, Folh1, Cnst, Dhcr7, Camk2g, Tmod1, Grn, Smim12, Tspan6, Cttn, Agpat3, Hs2st1, Timm23, Prnp, Eef1a1, Scn2b, Cd81, Carmil2, Akap12, Prkar2b, Slc20a2, Stx6, Dnajc16, Osbpl9, mt-Co3, Dgke, Mtx2, Palmd, Coq3, Sec61b, Mfn1, Copb1, Aldh3b1, Slc33a1, Ssr4, Tmem254c, Slc25a46, Cast, Lrrk2, Prmt1, Adcyap1r1, Rab11fip5, Plxna2, Map3k13, Cisd2, Nisch, Ryr3, Ranbp9, Rab3b, Cyp2d11, Ajm1, Micos10, Dagla, Ap1s1, Enpp6, Ndufs3, mt-Nd2, Nipal3, Sgtb, Atl1, Krt1, Sorcs1, Fkbp15, Sec11c, Itgb2, Gprin1, Tecpr1, Ndufa6, Sel1l, Gprc5b, Tmem237, Rhof, Cox7c, Neu1, Washc2, Slc44a2, Arhgap21, Kcnip2, Sorl1, Rbsn, Akap5, Cpne1, Gatm, Atp6v1g2, Ntng1, Acsl5, Akt2, Actbl2, Lman2, Acsbg1, Dag1, Syngr3, Osbpl1a, Lrrc8b, Jagn1, Ubxn4, Jup, Sdhc, Rdh13, Mtfp1, Als2, Mmgt1, Scd3, Palm2, Uqcr10, Cyp2s1, Kit, Ankh, Lama2, Gja1, Mtnd4, Gjc3, Slc27a1, Pnpla6, Stx4, Stard3nl, Rims2, Dmd, Msmo1, Slc12a4, Emc3, Tiam2, Ttyh1, Clvs1, Dmac2l, Diras1, Slc39a12, Wfs1, Rdx, Myorg, Rab5c, Syt11, Ptp4a2, Atg16l1, Kcnf1, Nlrx1, Adam17, Dnaja2, Pcsk2, Wdr81, Adcy8, Sdc4, Aph1a, Ephx1, Ppox, Lyst, Csmd3, Raf1, Atp6ap1, Clcc1, Vangl2, Armh3, Abhd17c, Hpcal1, Cds1, Sidt1, Slc4a4, Unc5a |
| Endosome | Mrc1, Pip4p1, Akt2, Rab11fip2, Rab5c, Syt11, Ptp4a2, Lrrk2, Osbpl1a, Adcyap1r1, Rab11fip5, Atg16l1, Nisch, Rab3b, Mmgt1, Dennd10, Dagla, Wdr81, Grn, Gja1, Fkbp15, Stx4, Arsa, Stard3nl, Optn, Washc2, Clvs1, Rbsn, Osbpl9, Sorl1, Akap5, Snx32, Abhd17c |
| Lamellipodium | Cttn, Rdx, Stx4, Dag1, Carmil2, Dmd, Tiam2, Evl, Nme2, Als2, Apbb1, Scyl3, Sh3bp1, Mtss2, Arpin |

**TABLE S4**

**The list of the proteins related to the top six KEGG pathway terms.**

| **KEGG pathway** | **Gene names of the related protein names** |
| --- | --- |
| Parkinson disease | Slc39a12, Mfn1, Psmb4, Mtnd4, Ndufa6, Lrrk2, Cox7c, Ndufa9, Ryr3, Sdhc, mt-Co3, Gnal, Psmd7, Camk2g, Uqcr10, Tuba8, Ndufs3, mt-Nd2 |
| Alzheimer disease | Aph1a, Slc39a12, Grin2d, Psmb4, Akt2, Mtnd4, Araf, Raf1, Ndufa6, Hsd17b10, Cox7c, Ndufa9, Ryr3, Adam17, Sdhc, Apbb1, mt-Co3, Psmd7, Uqcr10, Tuba8, Ndufs3, mt-Nd2 |
| Chemical carcinogenesis-reactive oxygen species | Ephx1, Akt2, Mtnd4, Araf, Sos2, Raf1, Ndufa6, Cox7c, Pten, Ndufa9, Sod2, Sdhc, mt-Co3, Uqcr10, Ndufs3, mt-Nd2 |
| Oxidative phosphorylation | Atp6v1g2, Ndufa9, Sdhc, Mtnd4, mt-Co3, Ndufa6, Lhpp, Uqcr10, Atp6ap1, Ndufs3, Cox7c, mt-Nd2 |
| Pathways of neurodegeneration-multiple diseases | Mfn1, Grin2d, Psmb4, Mtnd4, Araf, Prnp, Raf1, Ndufa6, Optn, Hsd17b10, Nefh, Lrrk2, Cox7c, Ndufa9, Ryr3, Sdhc, Als2, mt-Co3, Psmd7, Camk2g, Uqcr10, Tuba8, Ndufs3, mt-Nd2 |
| Ribosome | Rps5, Mrpl16, Mrps11, Mrpl9, Mrps10, Rpl23a, Rps15a, Rpl26, Rpl30, Rpl32, Rpl13, Rps20, Rpl6 |
